# Bitter taste cells in the ventricular walls of the murine brain regulate glucose homeostasis

**DOI:** 10.1038/s41467-023-37099-3

**Published:** 2023-03-22

**Authors:** Qiang Yu, Igor Gamayun, Philipp Wartenberg, Qian Zhang, Sen Qiao, Soumya Kusumakshi, Sarah Candlish, Viktoria Götz, Shuping Wen, Debajyoti Das, Amanda Wyatt, Vanessa Wahl, Fabien Ectors, Kathrin Kattler, Daniela Yildiz, Vincent Prevot, Markus Schwaninger, Gaetan Ternier, Paolo Giacobini, Philippe Ciofi, Timo D. Müller, Ulrich Boehm

**Affiliations:** 1 Department of Pharmacology, Center for Molecular Signaling (PZMS), Saarland University School of Medicine, Homburg, Germany; 2 Institute for Diabetes and Obesity, Helmholtz Diabetes Center, Helmholtz Zentrum München, Neuherberg, Germany; German Center for Diabetes Research (DZD), Neuherberg, Germany; 3 FARAH Mammalian Transgenics Platform, Liège University, Liège, Belgium; 4 Department of Genetics, Saarland University, Saarbrücken, Germany; 5 Univ. Lille, Inserm, CHU Lille, Laboratory of Development and Plasticity of the Postnatal Brain, Lille Neuroscience & Cognition, UMR-S1172, Lille, France; 6 Institute for Experimental and Clinical Pharmacology and Toxicology, Center of Brain, Behavior and Metabolism (CBBM), University of Lübeck, Lübeck, Germany; 7 Neurocentre Magendie - INSERM Unit 1215, University of Bordeaux, Bordeaux, France

**Keywords:** Neuroscience, Physiology

## Abstract

The median eminence (ME) is a circumventricular organ at the base of the brain that controls body homeostasis. Tanycytes are its specialized glial cells that constitute the ventricular walls and regulate different physiological states, however individual signaling pathways in these cells are incompletely understood. Here, we identify a functional tanycyte subpopulation that expresses key taste transduction genes including bitter taste receptors, the G protein gustducin and the gustatory ion channel TRPM5 (M5). M5 tanycytes have access to blood-borne cues via processes extended towards diaphragmed endothelial fenestrations in the ME and mediate bidirectional communication between the cerebrospinal fluid and blood. This subpopulation responds to metabolic signals including leptin and other hormonal cues and is transcriptionally reprogrammed upon fasting. Acute M5 tanycyte activation induces insulin secretion and acute diphtheria toxin-mediated M5 tanycyte depletion results in impaired glucose tolerance in diet-induced obese mice. We provide a cellular and molecular framework that defines how bitter taste cells in the ME integrate chemosensation with metabolism.

## Abstract (web summary)

The median eminence (ME) at the base of the brain controls body homeostasis. Here, the authors describe a functional tanycyte subpopulation at the ME which tastes the surrounding milieu, responds to metabolic signals and regulates glucose homeostasis.

## Introduction

The median eminence (ME) at the base of the brain is a central hub controlling body homeostasis^1,2^. Within this circumventricular organ, releasing hormones produced by neuroendocrine cells are secreted from axon terminals into the portal circulation and impinge onto hormone-secreting cells in the pituitary gland to regulate major hormonal body axes. Vice versa, peripheral hormones and other circulating cues can enter the brain by passive diffusion through the fenestrated capillaries within the ME^3,4^. This allows transduction of important signals to neighboring hypothalamic nuclei responsible for controlling body functions such as energy metabolism^5^ and reproduction^6^.

Tanycytes are specialized glial cells that line the walls of the third ventricle in the ME and are classified into four groups based on the position of their cell bodies along the ventricular wall^7–9^. α1 and α2 tanycytes are located along the ventricular surface of the ventromedial and arcuate nuclei, respectively. β1 tanycytes reside in the lateral extensions of the infundibular recess whereas β2 tanycytes line the floor of the ventricle^10^. Tanycytes thus have access to signals from multiple body compartments including the cerebrospinal fluid and the blood and have been implicated in regulating various physiological states^7^. However, while tanycytes have long been known to be essential for body homeostasis, individual signal transduction pathways in these cells are incompletely understood. Recent studies suggest that tanycytes respond to chemical stimuli including glucose, ATP, histamine, and acetylcholine^11^, probing the internal milieu of the organism and raising the possibility that these cells can be considered as taste cells within the brain. Taste cells within taste buds on the tongue express G protein-coupled receptors for bitter, sweet, and umami taste as well as TRPM5 (M5), a gustatory Ca^2+^-activated monovalent cation channel^12,13^. Upon tastant binding, taste receptors turn on a signaling cascade depending on the G protein gustducin which leads to PLCβ breakdown, production of DAG and IP3 and release of Ca^2+^ ions from the endoplasmic reticulum^14^. The cytosolic Ca^2+^ ions in turn open the M5 channel, leading to Na^+^ entry and depolarization of the plasma membrane^14^. M5-expressing cells were previously found in taste and chemosensory systems including the digestive tract^15^ and respiratory system^16^.

Here we identify a gustatory tanycyte subpopulation in the floor of the third ventricle. These cells express M5, bitter taste receptors and other key taste transduction genes such as the gustatory G protein gustducin. M5 tanycytes are functional taste cells and mediate bidirectional communication between the cerebrospinal fluid and the blood via processes extended towards diaphragmed endothelial fenestrations in the ME. M5 tanycytes also integrate metabolic signals and other hormonal cues and fasting leads to enhanced leptin signaling in these cells. Acute chemogenetic M5 tanycyte activation induces insulin secretion and acute diphtheria toxin-mediated M5 tanycyte depletion results in impaired glucose tolerance in mice. Our results provide a cellular and molecular framework for the integration of chemosensation with metabolism in the brain.

## Results

### Distinct tanycytes in the ME express the gustatory ion channel TRPM5

**Fig. 1:**
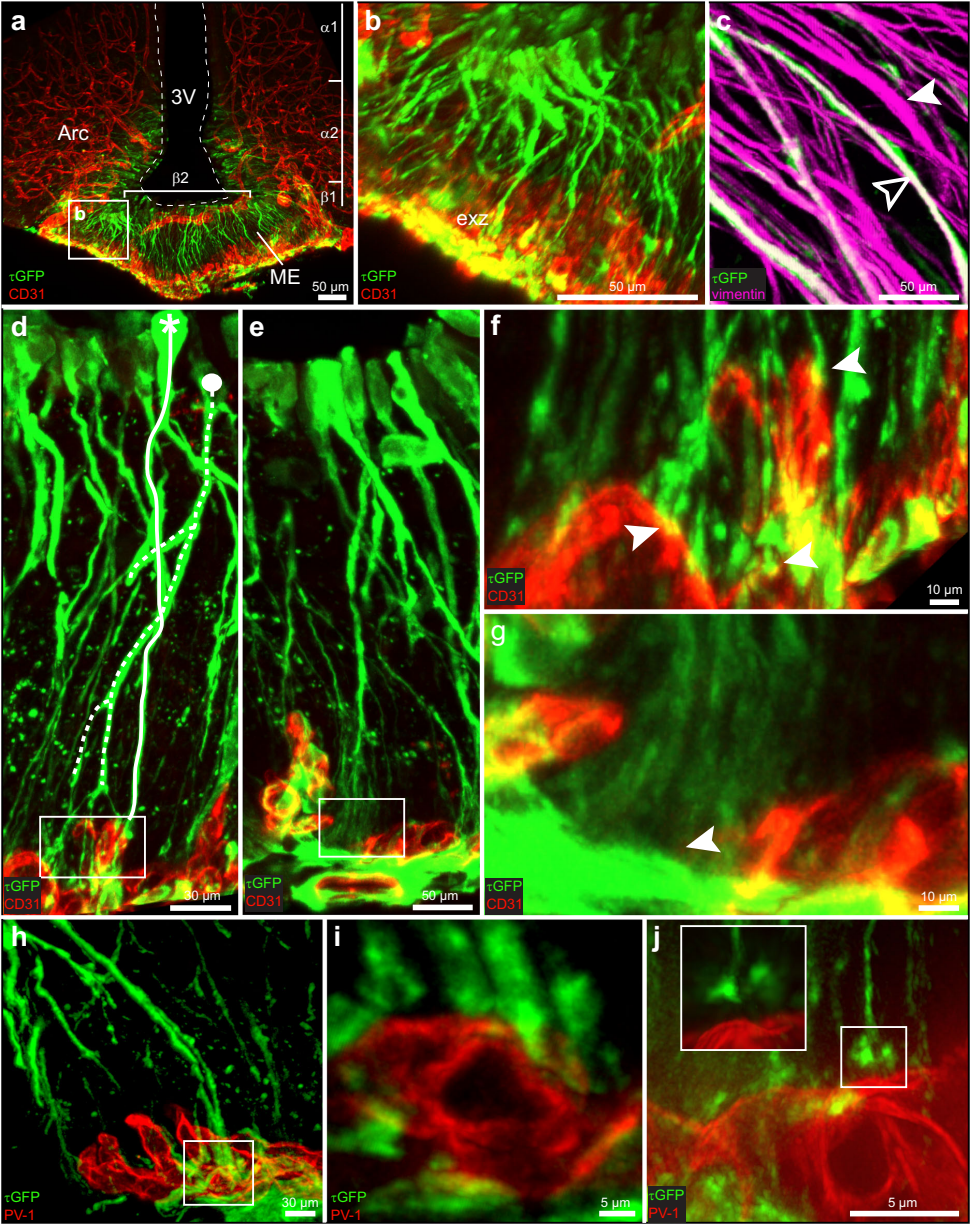
iDISCO-cleared and 3D-reconstructed images of the median eminence (ME) from M5-GFP mice immunolabeled for GFP (green, *n* = 3), the vascular endothelial marker CD31 (red), the endothelial permeability marker PV-1 (red), or the tanycyte marker vimentin (magenta). **a** M5 tanycytes are located in the floor of the third ventricle (3 V) at the level of the ventromedial arcuate nucleus (Arc) and ME. Tanycytic subpopulations based on previous classifications are indicated. **b** Enlarged from the box in **a**, shows the elongated morphology of the M5 tanycytes projecting to the ME external zone (exz) near the capillary vessels. **c** Not all vimentin-positive profiles (magenta) are simultaneously GFP-labeled (filled arrowheads), demonstrating that TRMP5 expression delineates a specific subpopulation of tanycytes (unfilled arrowheads). **d**-**g** Fine architecture of the M5 tanycytes. (**d**, detailed in **f**) Two individual M5 tanycytes (marked by solid and dotted lines) form branched processes with endfeet in close apposition (arrowheads, yellow overlay) to capillary vessels, visualized by CD-31 staining (red). (**e**, detailed in **g**) Other M5 tanycytes terminate at the parenchymal surface away from capillary vessels (arrowheads). **h**–**j** M5 tanycytes terminate near permeable microvessels (marked by PV-1 staining (red)), either with endfeet covering the microvessels (**h**, detailed in **i**) or seen at a distance using SIM microscopy (**j** and enlarged box). Scale bars: 50 µm (**a**‒**c**, **e**); 30 µm (**d**, **h**); 10 µm (**f**, **g**); 5 µm (**i**, **j**).

We first screened for cells expressing the TRPM5 ion channel, a key taste transduction gene, in the central nervous system (CNS) capitalizing on a recently established M5-GFP reporter mouse strain^15^. We found that M5 expression in the CNS is largely restricted to a small area in the hypothalamus, encompassing the arcuate nucleus and ME (Fig. 1a). Within this area, M5 cells are mainly confined to the walls of the third ventricle, indicating that these cells are tanycytes.

Based on their distribution, laterally facing the ventromedial arcuate nucleus and infundibular sulcus and medially the ME, most M5 tanycytes are of the β1- and β2-subtypes with some labeling also observed in α tanycytes (Fig. 1a). Their cell bodies constitute parts of the wall of the third ventricle and ventral projections from M5 β tanycytes reach the surface of the ME surrounding the blood vessels of the hypothalamo-hypophyseal portal plexus (Fig. 1a, b). Co-labeling for the tanycytic marker vimentin revealed that the M5 cells constitute a specific tanycyte subpopulation as many vimentin-positive profiles remained single-labeled (Fig. 1c). M5 tanycytes seem to be concentrated at the rostrocaudal mid-part of the ME (Supplementary Fig. 1a, b). They constitute ~5% of the total tanycyte population (4.060 M5 tanycytes out of a total of 73.590 tanycytes analyzed on sections prepared from 4 M5-GFP mice). We also observed M5 expression in the *pars tuberalis* (Supplementary Fig. 1c) and the choroid plexus (Supplementary Fig. 2).

### M5 β tanycytes have access to blood-borne cues

To trace the projections of individual M5 tanycytes, we used iDISCO tissue clearing with 3D reconstruction. We observed the classic morphology of successive branching into smaller processes forming brush-like distal profiles for the M5 β tanycytes (Fig. 1d-g, Supplementary Movie 1). Most of these projections form endfeet in close vicinity to capillary vessels (Fig. 1d, f). We also found M5 β tanycyte processes branching and terminating in capillary-free portions of the ME parenchymal surface (Fig. 1e, g). We next determined whether M5 β tanycytes terminate around permeable, fenestrated capillary vessels responsible for blood-to-brain exchanges in blood-brain barrier (BBB)-free areas, like the ME. Because endothelial fenestrations in the ME are diaphragmed, we generated an antiserum against the integral diaphragm protein PV-1, responsible for forming the fenestrations. We then performed immunostaining for PV-1 on brain sections prepared from the M5-GFP reporter mice and found that M5 tanycytic processes terminate at a variable distance from PV-1-positive capillaries (Fig. 1h, i) and as close as 0.5 µm based on structured illumination microscopy (SIM) measurements (Fig. 1j, Supplementary Movie 2). Taken together, these data suggest that the genetically defined M5 tanycytes transcend the previous classification of these cells and constitute a tanycyte subgroup. Because of the M5 β tanycyte access to blood-borne cues via the vessel fenestrations, where potential ligands might be sensed, we next investigated these cells using functional calcium imaging.

### Bidirectional calcium signal propagation in M5 β tanycytes

**Fig. 2:**
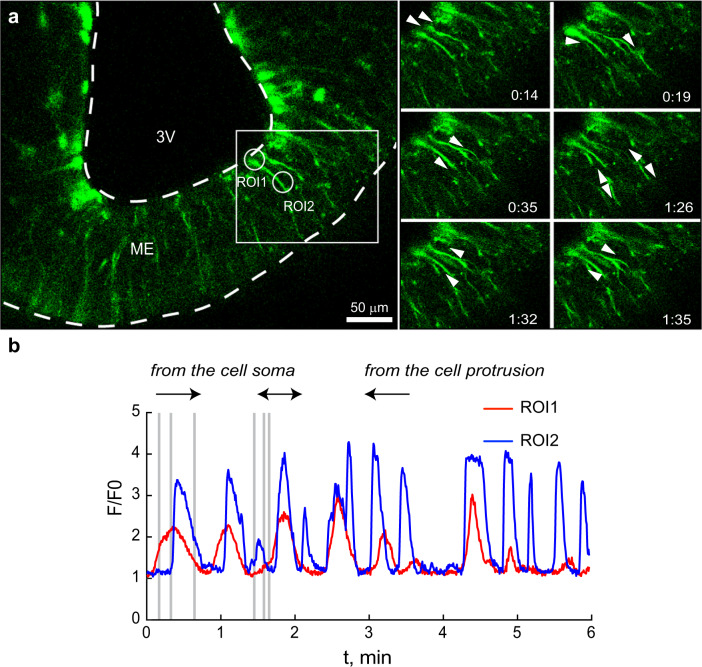
Bidirectional propagation of calcium transients within M5 tanycytes. **a** Images of a representative coronal section of the median eminence (ME) from a M5-GCaMP mouse showing spontaneous activity. Tanycytic cell bodies protrude from the third ventricle (3 V) wall to the external zone of the ME. Time frame series of the marked area (**a**, white rectangle) shows the propagation of the waves (see white arrows). **b** Normalized intensities of the GCaMP3 fluorescence from the cell body and the process (red trace and blue trace for ROI1 and ROI2 in a, respectively) measured as time course, demonstrate the calcium wave along the length of the tanycyte and its projection. Gray time marks in the graph correspond to the time frames in A in ascending order. The calcium wave propagates from the cell body to the end of the ME and back (black arrows) (spontaneous activity was observed during baseline activity in Ca^2+^ measurements, see for example Fig. 4b, *n* = 6 mice). Source data are provided as a Source Data file.

To functionally characterize the M5 tanycytes, we next analyzed acute ME slices prepared from mice expressing the calcium indicator GCaMP3 specifically in these cells. Using live cell confocal imaging, we observed spontaneous calcium signals in the M5 β tanycytes in both M5-GCaMP males and females. We detected a local increase in intracellular calcium in the cell bodies as well as in their emanating processes (Fig. 2a, Supplementary Movie 3). Within single M5 tanycytes, spontaneous calcium signals arose in sophisticated bidirectional waves (Fig. 2a, b). Specifically, within the M5 tanycytes found in one imaging plane (Fig. 2a, white rectangle), some calcium waves propagated from the cell bodies in the wall of the third ventricle towards the external zone of the ME spreading between tanycytic processes. Vice versa, other calcium waves in these cells propagated backwards towards the cell bodies (Fig. 2, time frames corresponding to the time points indicated as gray bars in the normalized intensity graph shown in b). During the spontaneous activity, we frequently observed a change in the direction of the calcium wave within the M5 tanycytes, as well as a rise of the intracellular calcium concentration in only one part of the cell (Fig. 2b, red and blue traces for the cell body and cell process, respectively, black arrows show the wave direction). Notably, the spontaneous activity of individual M5 tanycytes did not correlate with the activity of adjacent M5 tanycytes, since none of these exhibited a simultaneous calcium signal spreading from the cell bodies to the external zone of the ME (Fig. 2, time frames in a).

### M5 tanycytes are bitter taste cells

**Fig. 3:**
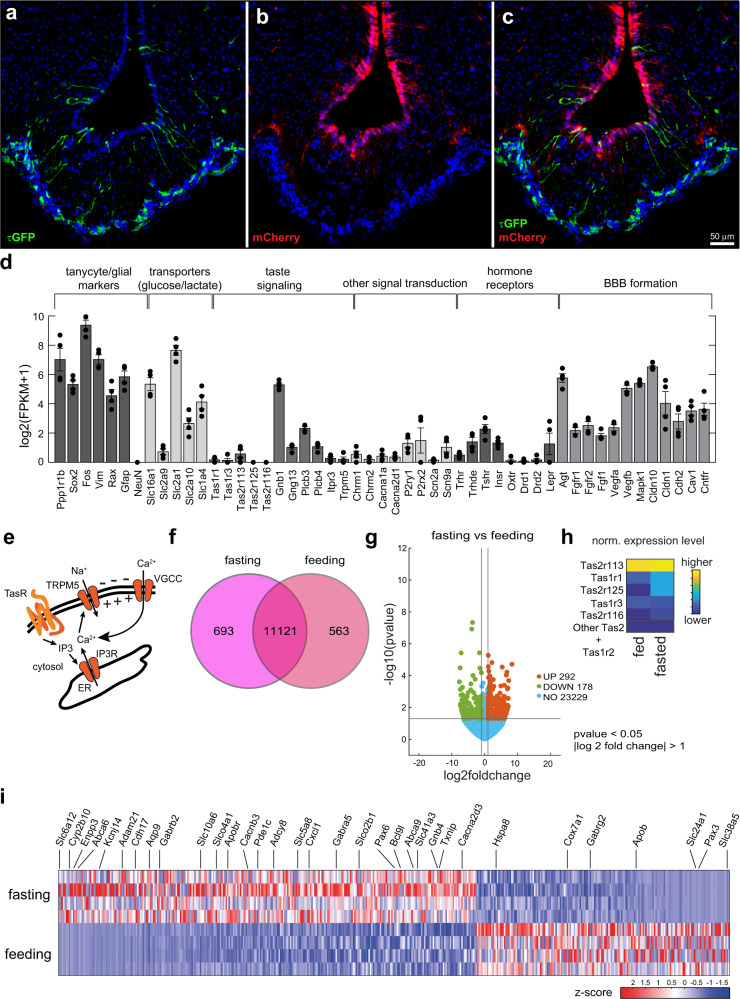
Expression profiling of M5 tanycytes. **a**–**c** AAV2/1 + 2-CAGS-mCherry virus was injected three weeks before FACS sorting of M5 tanycytes (*n* = 3). Expression of GFP in M5 tanycytes and *pars tuberalis* cells **a** and mCherry in tanycytes **b** before FACS sorting. **c** Note the colocalization of GFP and mCherry in the M5 tanycytes but not in the *pars tuberalis*. **d** Genes expressed in M5 tanycytes involved in different signaling pathways by RNA-seq (bar plot represents mean ± SEM from *n* = 4 different samples, individual log values shown for each gene). **e** Model indicates M5 activation after TasR activation. VGCC: voltage-gated calcium channels. **f** Venn diagram showing exclusively expressed genes. **g** Volcano plots showing the differentially expressed genes between feeding and fasting in M5 tanycytes (*p*-values were obtained via Wald test with DESeq2 package). **h** Heatmap shows expressed Tas receptors. **i** Heatmap of differentially expressed genes in fasting and feeding conditions. Highlighted genes are involved in cellular signaling and metabolism. Scale bars: 50 µm **a**–**c**. Error bars represent the standard error of the mean. Source data are provided as a Source Data file.

The anatomical position of the M5 tanycytes close to the ME would poise them to contribute to the control of energy metabolism by mediating communication between the blood, the CSF and neighboring hypothalamic areas including the arcuate nucleus. To test whether they adapt to extreme metabolic conditions such as fasting and further characterize these cells, we next analyzed the transcriptome of the M5 tanycytes isolated from both fed and fasted mice. To enrich for the M5 tanycytes and not co-purify the M5 pars tuberalis cells during fluorescence activated cell sorting (FACS) in M5-GFP reporter mice, we generated and injected AAV2/1 + 2-CAGS-mCherry virus into the lateral ventricle to mark the tanycytes three weeks before sacrifice. In this strategy, the M5 tanycytes -but not the M5 *pars tuberalis* cells- are labeled by both green and red fluorescence, allowing for dual fluorescence sorting (Fig. 3a–c). The basal hypothalamus was dissected from these adult male M5-GFP reporter mice, dissociated and then FACS sorting was performed to isolate GFP+ /mCherry+ cells, resulting in an enriched population of ~350 cells per mouse hypothalamus. Subsequent RNA-sequencing (RNA-seq) using an Illumina NovaSeq 6000 platform allowed detection of ~14.000 genes with fragments per kilobase of transcript per million mapped reads (FPKM) values >1 in the M5 tanycytes.

Classical tanycyte markers such as vimentin (*Vim*), transcription factors *Sox2* and *Rax* as well as protein phosphatase 1 regulatory subunit 1B (*Ppp1r1b*)^17^ were highly expressed in our sequencing data sets (Fig. 3d). While we found the glial marker GFAP in these cells, consistent with published data^10^, the neuronal marker NeuN was not detected, confirming the purity of the sorted cells. Several glucose and lactate transporters were detected (*Slc16a1, Slc2a9, Slc2a1, Slc2a10, Slc1a4*), consistent with a potential role of these cells in glucose sensing and metabolic status^18,19^. *Slc2a1* was previously reported to be highly expressed in β1 tanycytes and to a lesser extent in β2 tanycytes^20^. The M5 tanycytes expressed the purinergic *P2y1* receptor, suggesting that they may have a glucose-sensing mechanism similar to β-cells in the pancreas. We also identified key components of the taste signal transduction pathway (Fig. 3d), in particular taste receptors Tas2r113 and Tas2r125 (Fig. 3d), in the M5 tanycytes, suggesting that they represent ME taste cells. The observed absence or low expression for Tas1r2 and Tas1r3 respectively (Fig. 3d) would indicate that these cells seem to primarily detect bitter rather than umami or sweet taste. Activation of taste receptors would then be transduced via gustducin (*Gnb1*, *Gng13*) and propagated via the M5 channel and voltage-gated sodium and calcium channels (*Scn2a, Scn9a, Cacna1a, Cacna2d1*) (Fig. 3d), as previously established for tongue taste cells (Fig. 3e).

We also found metabolic hormone receptors such as those for leptin and insulin (*Lepr, Insr*) as well as receptors for other hormones including thyrotropin-releasing hormone and oxytocin (*Trhr, Oxtr*). Dopamine (*Drd*), muscarinic (*Chrm1, Chrm2*) and purinergic (*P2x, P2y*) receptor genes were also expressed (Fig. 3d). Furthermore, the M5 tanycytes express several genes implicated in BBB formation and the regulation of blood vessel permeability including vascular endothelial growth factors and angiotensinogen (*Vegfa, Vegfb, Agt*)^21^. β-tanycytes express claudin 1 (*Cldn1*), which is involved in the formation of a tight blood-CSF barrier^22^, and this was also expressed in the M5 subpopulation. Cadherin-2 (*Cdh2*) and caveolin-1 (*Cav1*), both involved in endocytosis and recycling, were also expressed. We also detected expression of genes underlying cell growth, adhesion, and proliferation including mitogen-activated protein kinase 1 (*Mapk1*), fibroblast growth factor 1 (*Fgf1*), fibroblast growth factor receptors (*Fgfr*) and ciliary neurotrophic factor receptor (*Cntfr*), possibly involved in the formation of the long protrusions and the blood vessel-touching endfeet extended by these cells, along with known factors of endfeet motility regulation such as transforming growth factors (TGFs) and semaphorins^7^.

### Fasting-induced reprogramming of M5 tanycytes

When comparing the gene expression patterns in cells isolated from fed and fasted animals, respectively, we found that while the majority of the genes displayed similar expression levels in these two conditions, a few genes, however, showed distinct expression patterns. Specifically, we found 11,121 genes commonly expressed in Venn analyses comparing both conditions (Fig. 3f). 563 genes were exclusively expressed in M5 tanycytes from fed mice, and 693 genes upon fasting. We identified 292 up-regulated genes and 178 genes that were down-regulated upon fasting (Fig. 3g). Functional analysis revealed that GO terms and pathways such as “receptor ligand activity”, “ATPase regulator activity” and “neuroactive ligand-receptor interaction” were more highly represented among these differentially expressed genes (Supplementary Fig. 3 and 4).

We found specific taste receptors to be differentially expressed under fed or fasted conditions. While Tas1r3 and Tas2r113 did not show expression differences between the two conditions, expression of both Tas1r1 and Tas2r125 increased upon fasting, with Tas2r125 being completely undetectable in fed mice. Tas2r116 was not detected in either condition (Fig. 3h). We identified several categories of genes which were differentially regulated between feeding and fasting (Fig. 3i). Genes participating in cellular signaling and signal transduction (*Gabrb2*, *Gabra5*, *Gnb4*, *Cacna2d3*, *Pde1c*) and several ABC transporters (*Abca6*, *Abca9*) were upregulated, as well as solute transporters (*Slc6a12*, *Slc41a3*), indicating an increased tanycytic activity upon fasting. Interestingly, genes participating in oxytocin signaling (*Kcnj14*, *Adcy8*)^23^ and also steroid transport (*Slc10a6*)^24^ were also upregulated upon fasting. In addition, genes related to lipid homeostasis including the receptor for low density lipoprotein (*Apobr*) were also differentially expressed between these two extreme nutritional conditions. Finally, genes controlling seasonal change sensation and hibernation such as thioredoxin-interacting protein (*Txnip*) were also regulated depending on energy status^25^, consistent with previous data suggesting that tanycytes may also play an important role in photoperiod acclimation^26^.

### Bitter taste signaling in the median eminence in vitro and in vivo

**Fig. 4:**
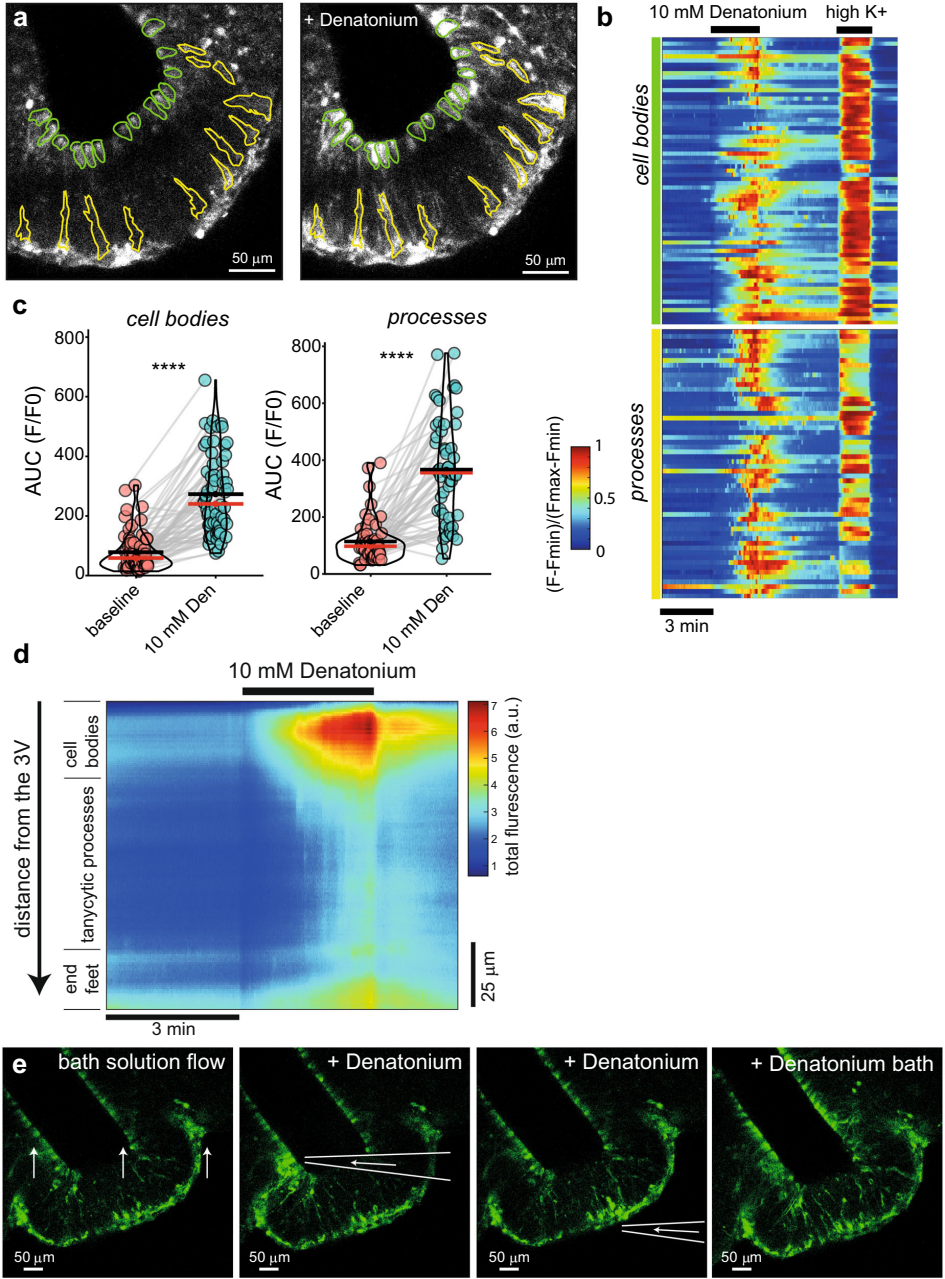
M5 tanycytes respond to bitter substances. **a** Calcium imaging of M5 tanycytes in the ME. Green and yellow ROIs represent cell bodies and tanycytic fibers respectively. Gray fluorescent pictures show the intensity changes after 10 mM denatonium application (representative observation from six experiments **b** Time course heatmap of the GCaMP3 normalized fluorescence change from the tanycytic cell bodies and processes, respectively (includes data from *n* = 6 mice, 12–24 weeks old, 2 males, 4 females). Black bars indicate the application of 10 mM denatonium and 30 mM K^+^ (high K^+^). **c** Area under the curve (AUC) of the normalized fluorescence intensities of the tanycytic cell bodies and processes. Every observed cell or process were collected for measurements: 3 min before denatonium (baseline) and 3 min after denatonium application (10 mM Den). Red lines are median, black lines are means for each group of measurements. Means were compared via two-sided t-test (****p = 1.23 10^−18^ for cell bodies and *p* = 2.06 10^−15^ for cell processes). **d** Heatmap showing fluorescence change through the ME (coronal sections from **a**, n = 6) from the third ventricle (3 V) wall (upper part) down to the fenestrated capillaries (lower part). Divisions represent apparent sections belonging to cell bodies, processes and end feet. Fluorescent intensity is color coded (color bar on the right, a.u.). Denatonium (10 mM) application is indicated by a black bar on the top, time axis direction from left to right (time scale 3 min, black bar). **e** Local application of denatonium via a pipette. Denatonium (250 mM) was applied to a constantly perfused coronal section (left panel) by bath solution (far right image panel), to the cell bodies (second image panel) and to the end feet (third image panel) (imaging was done two times per location on two different brain slices). Source data are provided as a Source Data file.

Since we identified key taste transduction genes such as Tas2R bitter taste GPCRs and gustducin subunits in the M5 tanycytes, we next studied functional bitter taste receptor signaling in these cells. To do this, we used confocal calcium imaging on acute coronal slices of the ME prepared from M5-GCaMP mice and bath-applied the Tas2r agonist denatonium (Fig. 4a). We found that denatonium evoked calcium elevations both in the M5 β tanycyte cell bodies and processes. Heat maps of the normalized fluorescence change (Fig. 4b, c) show that nearly all M5 β tanycytes were activated by denatonium.

We observed a time course of the calcium increase throughout the ME and found that the M5 cell bodies have higher increases in calcium compared to the processes and the M5 tanycytic endfeet (Fig. 4d). However, we did not see a clear time dependency between activation of the cell bodies and processes after the denatonium application. Furthermore, local application of denatonium via a patch pipette showed both activation of the cell bodies and the M5 tanycytic endfeet and processes (Fig. 4e). This suggests that the bitter taste receptors in the M5 β tanycytes are located both on the CSF and the capillary fenestration sides.

According to the classical taste transduction pathway^12^, TRPM5 activation occurs subsequent to binding of the bitter tastant to its receptor, depolarizing M5 tanycytes via Na^+^ entry, which in turn allows more calcium to enter the cell. To show the acute expression of TRPM5, we used stevioside, a potentiator of TRPM5^27^. We observed robust increases in calcium signaling in the M5 β tanycytes after stevioside (10 µM) application (Supplementary Fig. 5a, b). Similarly, intracerebroventricular injection of stevioside induced the activity marker c-Fos in M5 tanycytes (30.00% ± 6.27%, Supplementary Fig. 5c–f).

Consistent with this, responses to denatonium in the ME were significantly reduced in a TRPM5 knock-out background^13^ (Supplementary Fig. 6). Taken together, these data demonstrate TRPM5-dependent taste signaling in the M5 tanycytes in vitro and in vivo and confirm that these cells are functional bitter taste cells, capable of information exchange through the tanycytic barrier.

### Leptin activation of the floor of the third ventricle depends on M5 tanycytes

**Fig. 5:**
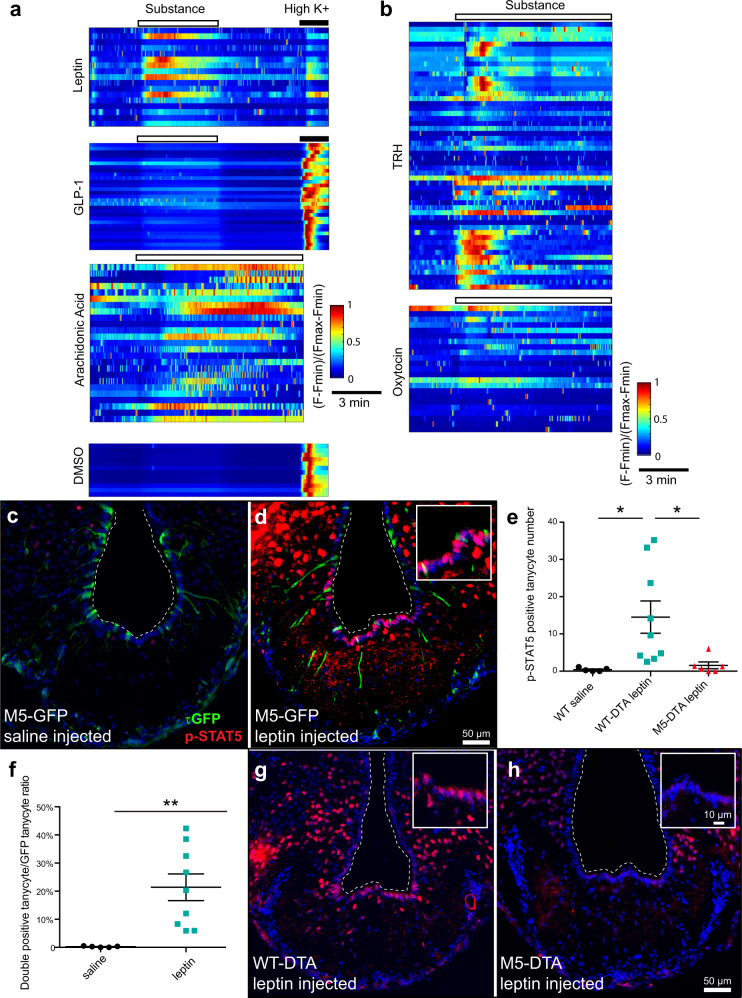
Hormonal and metabolic cues activate M5 tanycytes. **a** Representative heatmaps of the normalized fluorescence responses from the tanycytic cell bodies to metabolic blood-borne molecules. Application of a substance shown as an empty rectangle. The black bar indicates application of 30 mM K^+^. From upper to lower: Control application of DMSO (0.001%); Weak activation and moderate responses to GLP-1 (500 nM) and leptin (1 µg/ml), respectively, with comparison to the activation with Arachidonic acid (100 µM). Shown as cells pooled from 1 to 2 brain slices from 10 to 14 week old mice. **b** Representative heatmaps of Ca^2+^ responses, normalized as in a, upper: direct response to TRH (10 µM), lower: increase of cellular activity in response to Oxytocin (100 µM). Shown as cells pooled from 2-3 brain slices from 10 to 14 week old mice. **c-h** Phosphorylation of STAT5 in tanycytes is impaired in M5-DTA mice. p-STAT5 staining in overnight fasted M5-GFP mice injected with saline **c** or leptin **d**, quantification of p-STAT5 positive tanycytes and ratio of tanycytes double positive for both p-STAT5 and GFP **e**, **f**, WT-DTA (DTA-) **g**, and M5-DTA (DTA + ) **h** mice injected with leptin (WT saline *n* = 5 **e**, WT-DTA leptin *n* = 9 **e**, **g**, M5-DTA leptin *n* = 6 **h**, **e**, M5-GFP saline *n* = 5 **c**, **f**, M5-GFP leptin *n* = 9 **d**, **f**, data analyzed with 2-tailed t-test). WT saline vs WT-DTA leptin *p* = 0.0346, WT-DTA leptin vs M5-DTA leptin *p* = 0.0328, saline vs leptin *p* = 0.0067. Note the p-STAT5 signal in tanycytes in the M5-GFP and WT-DTA mice which was lost in M5-DTA mice. Scalebars: 50 µm (overview), 10 µm (inset). Error bars represent the standard error of the mean. Source data are provided as a Source Data file.

We next tested, whether metabolic and other hormonal cues activate M5 tanycytes in vitro. Upon application of leptin, M5 tanycytes robustly responded with an increase in intracellular calcium (Fig. 5a). In comparison, glucagon-like peptide 1 (GLP-1), another satiety hormone, was much less potent in triggering calcium responses in these cells. The M5 tanycytes also showed a strong and continuous reaction to the food supplement arachidonic acid. Furthermore, releasing hormones such as TRH as well as oxytocin triggered calcium signals (Fig. 5b), demonstrating that the M5 tanycytes respond to different hormonal cues.

To identify the signaling pathway activated by leptin in M5 tanycytes, we injected leptin into the cerebral ventricle and then immunostained for signaling components downstream of the leptin receptor in the arcuate nucleus and in nearby tanycytes. We detected nuclear phosphorylated signal transducer and activator of transcription 5 (p-STAT5) both in cells in the arcuate nucleus and in tanycytes 30 min after leptin injection, which was essentially absent in saline-injected controls (Fig. 5c, d). Some of the p-STAT5-immunostained tanycytes were M5 cells (21.4% ± 4.7%), raising the possibility that these cells are transcriptionally reprogrammed upon leptin stimulation via p-STAT5 signaling (Fig. 5e, f). Next, we tested leptin signaling across the ME in animals lacking M5 tanycytes. To do this, we generated mice which express diphtheria toxin A chain (DTA) selectively in M5 cells. DTA inhibits translation by catalyzing the ADP ribosylation of the eukaryotic elongation factor 2, resulting in cell death and thus depleting the M5 cells^28^. Notably, loss of phosphorylated STAT5 in M5-DTA mice was not restricted to the M5 tanycyte population. Instead, the p-STAT5 immunosignal was absent in all tanycytes in these animals (Fig. 5g, h). Ablation of M5 tanycytes did not lead to a leaky ME barrier as we did not observe a statistically significant difference in transvascular Evans Blue diffusion or vimentin staining between WT and ablated animals (Supplementary Fig. 7 and 8). We also observed a calcium response to 100 µM ATP both in WT and ablated animals as a functional control (Supplementary Fig. 7). Taken together, these data suggest that leptin activation of p-STAT5 signaling in tanycytes depends on the M5 subpopulation. Since these cells only constitute a small subpopulation of all tanycytes (Fig. 5), our data indicate complex interactions between different tanycyte subpopulations possibly depending on TRPM5 activation by an increase in intracellular calcium and raises the possibility that these cells influence metabolism in vivo.

### Impaired glucose tolerance upon acute diphtheria toxin-mediated M5 tanycyte depletion

**Fig. 6:**
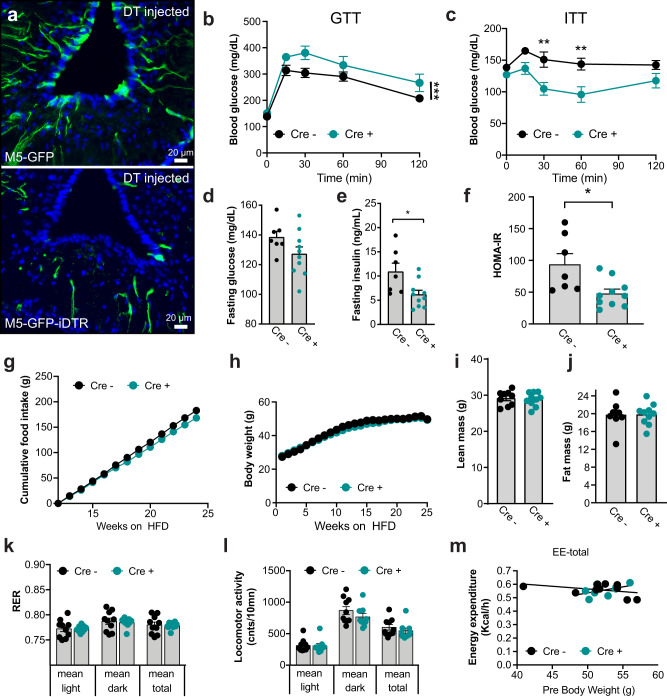
Acute M5 tanycyte ablation impairs glucose tolerance. **a** M5-GFP mice and M5-GFP-iDTR mice were bilaterally intra-hypothalamically injected with 0.5 ng diphtheria toxin and perfused 7 days later. Note the ablation of the M5 tanycytes (*n* = 3). Scale bar: 20 µm. **b** Glucose tolerance test (GTT) at age of 30 weeks (*n* = 10, *p* = 0.0004). **c**–**e** Insulin tolerance test (ITT) (*p* < 0.01 for 30 and 60 min), fasting glucose (*p* = 0.1096), and fasting insulin (*p* = 0.0181) in 48 weeks old WT mice and mice with DT-mediated M5 tanycyte depletion (Cre + ) (Cre- *n* = 7, Cre+ n = 10). HOMA-IR **f** at the age of 48 weeks (Cre- n = 7, Cre+ *n* = 10, *p* = 0.0123), food intake **g** (Cre- *n* = 4, Cre+ *n* = 7), body weight (**h**) (Cre- n = 11, Cre+ *n* = 10), body composition (**i**, **j**) at the age of 33 weeks (Cre- *n* = 9, Cre+ n = 10), respiratory exchange ratio (RER), locomotor activity and total energy expenditure **k**–**m** in 33 weeks old WT and KO mice (Cre^-^ *n* = 10, Cre^+^ *n* = 9). Data represent means ± SEM. Asterisks indicate ** *p* < 0.01. Longitudinal data **b**, **c**, **g**, **h** were analyzed using 2-way ANOVA with time and genotype as co-variables and Bonferroni post-hoc analysis for individual time-points. Bar graphs **d**–**f**, **i**–**l** were analyzed us**i**ng 2-tailed t-test. Data in **m** were analyzed using ANCOVA with body weight as co-variate. Source data are provided as a Source Data file.

To test this hypothesis and unravel the physiological function of the M5 tanycyte population, we acutely ablated these cells using diphtheria toxin (DT). The diphtheria toxin receptor (DTR) promotes translocation of DT into the cytoplasm resulting in the inhibition of protein synthesis and subsequently cell death. We found that 0.5 ng DT injected bilaterally into the arcuate nucleus of male mice, in which DTR expression is restricted to M5 cells (M5-DTR), efficiently depleted the M5 tanycyte population (Fig. 6a), but not the M5 pars tuberalis or choroid plexus cells (Supplementary Fig. 9). Interestingly, when chronically fed with a HFD (but not when on normal chow, Supplementary Fig. 10a, b), mice with DT-mediated M5 tanycyte depletion showed impaired glucose sensitivity (Fig. 6b) despite improved sensitivity to insulin 18 weeks later (Fig. 6c) with non-statistically lower baseline glucose (Fig. 6d) but decreased levels of insulin (Fig. 6e). Mice with DT-mediated M5 tanycyte depletion also showed a lower HOMA-IR value after M5 tanycyte depletion (Fig. 6f). Food intake was unchanged between M5 tanycyte-depleted mice and controls (Fig. 6g). The animals did not show alterations in body weight (Fig. 6h) or body composition (lean and fat tissue mass, Fig. 6i, j). No difference was seen in respiratory exchange ratio (Fig. 6k), locomotor activity (Fig. 6l) or energy expenditure (Fig. 6m, Supplementary Fig. 10c, d). Taken together, these data demonstrate that acute M5 tanycyte ablation impairs glucose tolerance after high fat diet feeding but increases insulin sensitivity, reduces insulin secretion and produces a non-statistically significant lower baseline glucose.

### Chemogenetic M5 tanycyte activation promotes insulin release

**Fig. 7:**
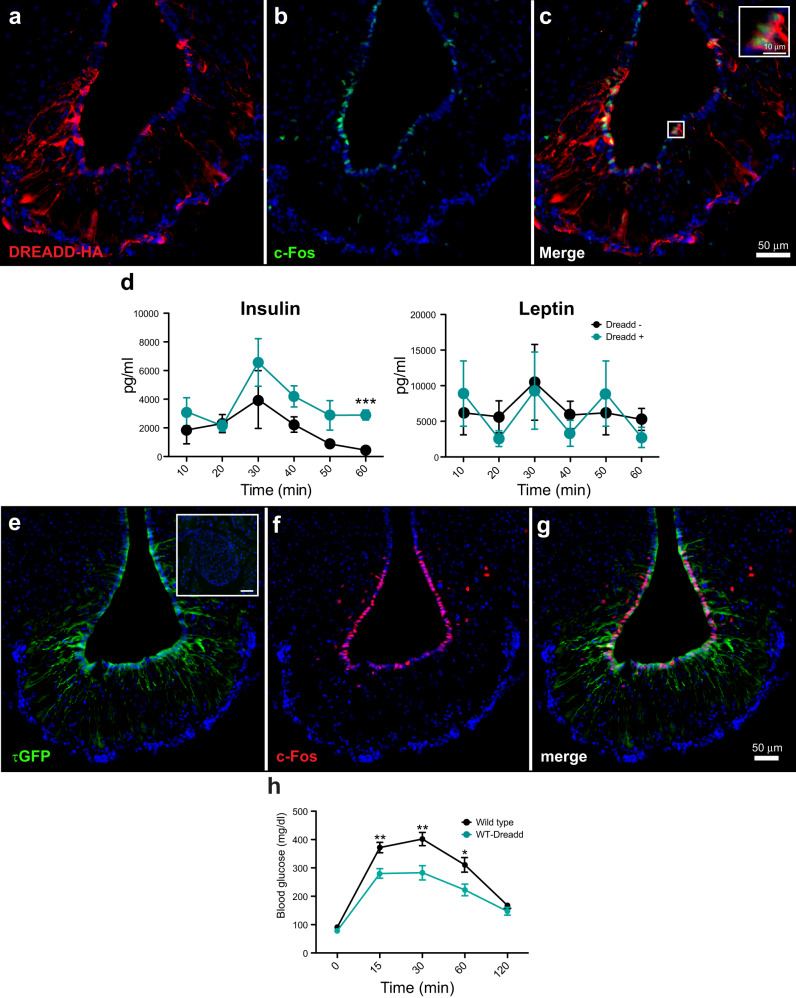
Acute M5 tanycyte activation promotes insulin release. **a**–**c** M5-DREADD mice were injected with 2 μg CNO into the third ventricle and perfused 1 h later. M5 tanycytes were detected via an anti-HA tag antibody. Note the colocalization of c-Fos and HA tag signal. Scalebar: 50 µm (overview), 10 µm (inset) (result replicated in *n* = 5 mice). **d** Insulin release after M5 tanycyte activation. Mice cheek blood obtained at various time points after third ventricle injection of CNO both in WT and M5-DREADD mice. Serum levels of insulin and leptin were tested via Luminex MAGPIX System (*n* = 4). Note that insulin release increased significantly in M5-DREADD mice compared with WT, data analyzed with 2-tailed t-test. Asterisks indicate *** *p* < 0.001. **e**–**g** Tanycyte activation via CNO i.p. injection three weeks after AAV-DIO2-Cre virus injected into WT-GFP/DREADD mice. Note the colocalization of GFP and c-Fos signal. Inset: GFP was not detected in the pancreatic islet. Scalebars: 50 µm, (*n* = 3). **h** Glucose tolerance test between WT-DREADD and WT mice i.c.v. injected with AAV-DIO2-Cre virus (wild type *n* = 8, WT-DREADD *n* = 5). Data were analyzed using 2-tailed t-test. Asterisks indicate * *p* < 0.05, ** *p* < 0.01. Error bars represent the standard error of the mean. Source data are provided as a Source Data file.

To unravel the mechanism behind the decreased levels of insulin and impaired glucose tolerance observed in M5 tanycyte-ablated mice, we used a complementary experimental strategy and acutely activated the M5 tanycyte population in mice which express HA-tagged designer receptors exclusively activated by designer drugs (DREADD) selectively in M5 cells (M5-DREADD). To validate these mice, 2 μg CNO, a specific DREADD activator, was injected into the third ventricle and these were perfused 1 h later. Co-staining of HA-tag and c-Fos immunoreactivity verified selective activation of the M5 tanycytes (Fig. 7a–c). Blood was collected at different time points and the hormone levels were tested via Luminex. After CNO injection, we did not observe differences in circulating leptin levels from 10 to 60 min. However, while insulin levels showed no differences between WT and M5-DREADD mice at 10 and 20 min after injection, M5-DREADD mice displayed increased insulin release between 30 min and 60 min after CNO administration (Fig. 7d). Over the same time period, we did not observe fluctuation of pituitary hormones following activation of M5 tanycytes (Supplementary Fig. 11), indicating that activation of these cells specifically promotes insulin release. To further corroborate these findings, we injected an AAV-DIO2-Cre virus (which expresses Cre recombinase under transcriptional control of type II iodothyronine deiodinase (Dio2) promoter in all tanycytes^29^), into the cerebral ventricle of WT-DREADD/GFP mice. Three weeks after injection, expression of GFP and thus DREADD was found in the tanycytes but not in the pars tuberalis (Fig. 7e). Intraperitoneal injection of CNO induced c-Fos expression in tanycytes and not in the pars tuberalis (Fig. 7e–g). We then repeated the glucose tolerance test following i.p. CNO injection and found an improvement in glucose tolerance in virus-injected WT-DREADD animals when compared to virus-injected control animals lacking the DREADD allele (Fig. 7e–h). Taken together, these data strongly suggest, that the M5 tanycytes are responsible for the observed GTT phenotype and promote insulin release.

**Fig. 8:**
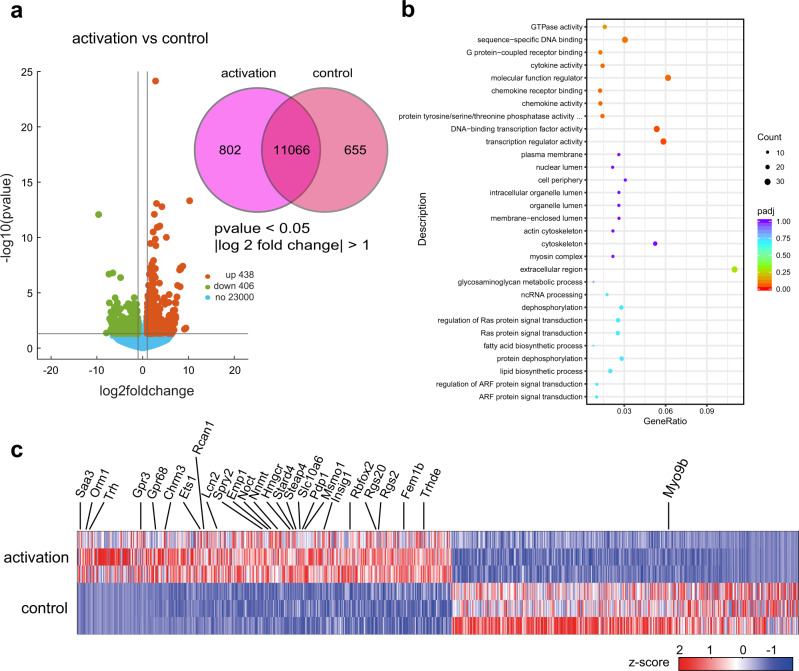
M5 tanycyte activation leads to differential regulation of metabolism-related genes. **a** (right) Venn diagram showing exclusively expressed genes and (left) Volcano plot illustrating genes differentially regulated in activated M5 tanycytes compared with controls by RNA-seq (*p*-values were obtained via Wald test with DESeq2 package, *n* = 3 samples). **b** Scatter Plot of GO terms enriched from upregulated genes in activated TRPM5 tanycytes. The color and size of the dots are scaled with respect to padj value and the number of the differentially expressed genes, respectively. For **a** *p*-values were obtained via Wald test with DESeq2 package. For **b** padj were obtained via hypergeometric test, and FDR correction was done by Benjamini and Hochberg method. **c** Heatmap of differentially expressed genes in activated vs control M5 tanycytes. Highlighted genes are predominantly involved in metabolism. Source data are provided as a Source Data file.

To understand this phenomenon, we performed transcriptomic analysis on the activated M5 tanycytes (Fig. 8). We found that 438 genes were significantly upregulated and 406 genes were downregulated in chemogenetically activated M5 tanycytes when compared with controls (Fig. 8a Volcano plot). After M5 tanycyte activation, we found up-regulation of several genes related to metabolism including *Orm1*, which was reported to activate the JAK2-STAT3 pathway upon binding to the leptin receptor and improve glucose and insulin tolerance^30^. Some genes related to cholesterol metabolism were also up-regulated in activated M5 tanycytes such as *Hmgcr*, *Stard4*, *Slc10a6*, *Msmo1*, *Insig1* and *Rbfox2*, indicating a function of M5 tanycytes in maintaining lipid homeostasis^31–36^. In addition, membrane proteins including *Emp1* which colocalize with tight junction proteins and regulate blood brain barrier function were also up-regulated following M5 tanycyte activation^37^. Interestingly, we also identified increased expression of a selection of genes related to insulin production and glucose regulation upon stimulation of the M5 tanycytes, such as *Pdp1*, *Rgs2* and *Fem1b*^38–40^. Taken together, these sequencing results are consistent with a role of M5 tanycytes in the regulation of metabolism within the mouse.

## Discussion

We have identified a gustatory cell type in the floor of the third ventricle that integrates chemosensory and hormonal cues and provides a selective means of bidirectional communication between the CSF and capillary system. Based on their polarized morphology and location, the chemosensory M5 cells are poised to mediate communication between central and peripheral environments and subsequently influence energy metabolism^7^. Despite previous studies suggesting important roles of tanycytes in the control of body homeostasis, functional signaling pathways in these cells are, however, only now beginning to emerge. The M5 cells in the ME constitute a small subpopulation of the tanycytes, demonstrating functional specialization within the tanycyte population and transcending the previous classification of these cells based on anatomical markers.

We found spontaneously active M5 tanycytes with some cells displaying repetitive signaling patterns during the 30 min measurements, likely reflecting nerve terminal activity preserved in the acutely prepared ME slices. We rarely observed calcium waves spreading between adjacent tanycytes, in contrast to a previous report^41^. Bidirectional propagation of the calcium signals between the soma and the tanycytic processes indicates communication between the CSF and the capillary portal plexus. Calcium responses to specific hormones, such as TRH, may reflect a modulatory role by M5 tanycytes on fenestration accessibility based on hormone status^42^.

While first described on the tongue^43^, taste cells have also been identified in extraoral tissues including the intestinal tract, stomach, and lung^44^ demonstrating that taste receptor expression is not restricted to the gustatory system. Tas2r taste receptors differ widely in their tuning breadth, ranging from broadly tuned receptors that recognize numerous compounds to receptors that are narrowly tuned and detect only single or very few compounds^45^. We identified the narrowly tuned bitter taste Tas2r113 and Tas2r125 receptors as well as other key components of the classical taste transduction pathway in M5 tanycytes and confirmed the responses of these cells to bitter substances using functional imaging. In a heterologous cell culture expression setup, Tas2r113 could be activated with phenylbutazone, a synthetic drug compound and Tas2r125 with phenyl-β-d-glucopyranoside and umbelliferone, both of which are natural plant substances without obvious murine counterparts^45^. The identification of the endogenous ligands of these two and other Tas2 receptors in the M5 tanycytes remains a task for future studies. It is however tempting to speculate that bitter sensing by the chemosensory M5 tanycytes as part of the tanycytic barrier in the ME may protect against the access of toxic molecules from the capillary portal plexus.

Metabolic phenotyping revealed impaired glucose tolerance following M5 tanycyte ablation coupled with longtime (>20 weeks) high fat diet feeding, as well as increased insulin sensitivity and reduced insulin concentrations after 18 weeks, despite unchanged body weight and fat mass. In stark contrast, two previous studies reported unchanged (or even somewhat improved) glucose tolerance and increased body weight and visceral fat mass after ablation of all β1 and β2 tanycytes^46^ or knocking out of glucokinase expression in tanycytes which subsequently induces apoptosis in these cells^47^. One obvious difference between the two studies is that here, we selectively remove the M5 tanycyte subpopulation. Ablation of all (VEGFa-expressing) β1 and β2 tanycytes both in the floor as well as in the ventral lateral walls of the ME and third ventricle seems sufficient to open up the CSF barrier towards the arcuate nucleus^46^, possibly explaining the differences in body weight in the two distinct experimental approaches. Taken together, these data highlight the necessity to continue to identify and characterize functionally distinct tanycyte subpopulations beyond the classifications based on anatomical markers.

How can impaired glucose tolerance in the absence of M5 tanycytes be explained? The brain senses blood glucose levels through vein sensors located in close proximity to the capillary portal plexus in the ME. We found that the M5 tanycytes have a close connection to the fenestrated capillaries and cover the latter with their endfeet. As the functional plasticity of endfeet is a Gq-dependent process^42^, the M5 channel might underlie opening of voltage-gated channels increasing the intracellular calcium concentration to initiate endfoot plasticity. An absence of M5 tanycytes may thus impair hypothalamic barrier functioning and feedback integration. Consistent with this, we found the M5 tanycytes to express genes important in BBB formation. An alternative, but not exclusive, hypothesis is that because M5 tanycytes are highly sensitive to leptin, they may be tightly involved in the shuttling of circulating leptin into the hypothalamus, a process that we have recently shown to play a key role in the regulation of pancreatic β-cell function^48^, certainly via the melanocortin system^49^.

M5 tanycytes do not express the Tas1r2 sweet taste receptor, suggesting that these cells do not sense glucose via this mechanism. The M5 tanycytes mainly express the glucose transporter Glut1 (*Slc2a1*), but not Glut 2 and very little Glut3. These transporters have different Km values (Glut1 6.9 mM, Glut2 11-17 mM, Glut3 1.4 − 1.8 mM)^50^. Glucose levels in the interstitial fluid of the brain are lower (~0.7 mM in fasting condition and 1.4 mM after feeding)^51^ than those in peripheral blood (5.8 mM in fasting condition and 7.7 mM after feeding), indicating that the M5 tanycytes likely do not sense glucose from interstitial fluid. Instead, their endfeet close to the fenestrated blood vessels in the ME possibly detect high glucose levels in the peripheral blood and transmit this information to the brain^19^. The M5 tanycytes could also contribute to increased cerebral blood flow in response to hypoglycemia^52^.

We demonstrate that M5 tanycytes express the leptin receptor (LepR) and respond to leptin. Furthermore, leptin activation of p-STAT5 signaling in the floor of the third ventricle (which typically activates all tanycytes) seemed to depend on the M5 subpopulation, possibly through tanycyte intercommunication via connexin 43^19,53^. The lack of leptin signaling in the ME could also explain the impaired glucose tolerance phenotype in the acute M5 tanycyte ablation model. Consistent with this, previous studies showed that mice with a deletion of the LepR in tanycytes developed impaired glucose tolerance associated with an alteration in insulin release^48^. GLP-1 had only mild effects on calcium responses in M5 tanycytes. While GLP1R has recently been shown to be expressed in tanycytes using highly sensitive in situ hybridization approaches^54^ and immunohistochemistry^55^, our RNA sequencing data did not detect GLP-1 receptor transcripts, however, the expression could be below our detection threshold. We found signal transduction pathway components resembling other extraoral M5 chemosensory cells such as those in the small intestine and stomach, which emphasizes the functional role of M5 tanycytes in chemosensation^12,56^. M5 is also expressed in pancreatic β-cells and Trpm5-/- mice display decreased glucose tolerance^57–59^.

We also identified key components of the hypothalamic-pituitary-thyroid axis including thyrotropin-releasing hormone degrading enzyme (Trhde), thyrotropin-releasing hormone receptor (Trhr), and thyroid stimulating hormone receptor (Tshr) in M5 tanycytes (Fig. 3d), raising the possibility that these cells could modulate glucose homeostasis via this signaling pathway^42^. Tanycytes have previously been shown to regulate T3 access to the paraventricular nucleus of the hypothalamus (PVN), where elevated T3 increases glucose production via sympathetic connections between the PVN and the liver^60^. Glut1 and 4 expression is upregulated after T3 treatment in different tissues, increasing glucose uptake^61,62^.

M5 tanycytes also express 11β-hydroxysteroid dehydrogenase type 1 (Hsd11b1), an enzyme controlling glucocorticoid production and thus gluconeogenesis^63^. Glucocorticoids have previously been reported to decrease Glut4 expression^64^.

Impaired glucose tolerance upon M5 tanycyte depletion was accompanied by improved insulin sensitivity but decreased plasma insulin levels. This suggests hypoinsulinemia consistent with lower fasting insulin in these animals (Fig. 6e). The autonomic nervous system plays an important role in controlling pancreatic islet function^65^. Excessive reactive oxygen species (ROS) production in POMC neurons in the arcuate nucleus of Mitofusin 1 knockout mice enhanced sympathetic activity in the pancreas and impaired glucose-stimulated insulin secretion (GSIS) without changes in body weight, and intracerebroventricular (i.c.v.) administration of ROS scavengers normalized GSIS in these animals^66^. Recently, tanycytes have been shown to regulate lipid homeostasis by palmitate uptake promoting ROS production, engaging the p38-MAPK pathway and resulting in FGF21 production^67^. In addition, M5 tanycyte-specific activation promoting insulin secretion in M5-DREADD mice (Fig. 7d) also resembles the acute effect of ROS scavengers on GSIS in the *POMCMfn1KO* mice^66^. Whether M5 tanycytes influence ROS level in the arcuate nucleus will need to be addressed in future studies. A recent study has shown, that tanycyte activation can evoke depolarization in both NPY and POMC neurons via ATP release, inducing acute hyperphagia^41^. Which types of neurons are contacted by M5 tanycytes and the downstream effects of M5 tanycyte stimulation on these cells will need to be addressed in future studies.

Finally, an alternative explanation for the impaired glucose tolerance in the ablated mice may be that this perturbates communication between the brain and pancreatic beta cells. The hypothalamus communicates through the NTS with the pancreatic islets and the islets are sending information to the brain through the vagus nerve NTS connection^68^ to maintain the glucose homeostasis of the body.

## Methods

### Generation of the Rosa26-NLSiRFP720-2A-Gq (DREADD) knock-in mice

DREADD mice were generated by homologous recombination in mouse embryonic stem (ES) cells using a targeting construct designed to insert a CAGS promoter (CMV enhancer plus chicken β-actin promoter)-driven NLSiRFP720-2A-Gq receptor (DREADD receptor) within the first intron of the *Rosa26* gene locus. This encodes both an infrared fluorescent protein, which is directed to the cell nucleus, and a Gq-coupled receptor, which can be specifically activated by CNO administration. To ensure that this expression is Cre-dependent, floxed strong transcriptional stop signals (three SV40 polyA signals) are present in such a way that the CAGS promoter can only drive expression following Cre-dependent removal of the stop signals. Correct insertion of the NLSiRFP720-2A-Gq receptor construct was verified using Southern blot analysis as follows. DNA was extracted from tail tip biopsies or ES cells using lysis buffer containing 0.1 mg/mL proteinase K (1 mg/mL was used for extraction from ES cells). Following extraction, genomic DNA was digested overnight with *EcoR*I and run on a 0.7% agarose gel, then transferred to a nylon membrane by capillary transfer and screened by hybridization of a 491 bp ^32^P-labeled probe complementary to sequences located 5′ to the 5′ homology arm of the targeting construct. Probe hybridization produces a 15.6-kb band from the wild-type allele, whereas the correctly targeted allele generates a 5.8-kb band. Correctly targeted ES cells were injected into C57BL/6 J blastocysts to generate male chimeras that were backcrossed to C57BL/6 J females to produce heterozygous *Rosa26*-NLSiRFP720-2A-Gq mice. Mice were then further crossed to produce a homozygous colony. Mice were maintained on a mixed genetic background of 129 S × C57BL/6 J. The genotypes of the *Rosa26*-NLSiRFP720-2A-Gq mice were confirmed by PCR using the primer sequences: 5-GGAAGCACTTGCTCTCCCAAAG-3′ (common forward primer); 5′-GGGCGTACTTGGCATATGATACAC-3′ (DREADD allele reverse primer) and 5′-CTTTAAGCCTGCCCAGAAGACTC-3′ (wildtype allele reverse primer). WT offspring were confirmed by the presence of a single band of 256 bp. For the *Rosa26*-NLSiRFP720-2A-Gq allele, heterozygous offspring gave two products of 256 and 495 bp, whereas homozygous offspring were identified by the presence of one band at 495 bp.

### Mice

Animal care and experimental procedures were approved by the animal welfare committee of Saarland University, the Regierung Oberbayern and the French Ministry of National Education, Higher Education and Research (APAFIS#2617-2015110517317420 v5) and were performed in accordance with their established guidelines. Experiments were designed based on accepted standards of animal care and all efforts were made to minimize animal suffering. Mice were kept at an ambient temperature of 22 ± 2 °C with constant humidity (45–65%) and a 12 h/12 h light/dark cycle. For calcium imaging experiments both male and female mice were used. For all other experiments, male mice were used. Mice were kept under a standard light/dark cycle with food and water *ad libitum*. To label TRPM5-expressing cells, we used the TRPM5-IRES-Cre (M5)^15^ knock-in mouse strain crossed with eROSA26-τGFP (GFP)^69^, eROSA26-GCaMP3 (GCaMP)^70^ (generously provided by Dr. D. Bergles, Johns Hopkins University, Baltimore), ROSA26-DTA (DTA)^28^ or eROSA26-DREADD (DREADD) animals. In the resulting M5-reporter/effector mice, Cre recombinase is expressed under control of the *Trpm5* promotor. Cre-mediated recombination results in the removal of a strong transcriptional stop cassette from the *ROSA26* locus and subsequent constitutive reporter expression in TRPM5 cells. Mice were kept in a mixed (129/SvJ and C57BL/6 J) background. All animals used in this study were heterozygous for the TRPM5-IC and the eR26-reporter/effector alleles, respectively. To deplete M5 tanycytes, M5-GFP animals were crossed with ROSA26-iDTR (iDTR)^71^ mice to generate offspring in which, following Cre-mediated recombination, τGFP and the diphtheria toxin receptor are expressed in TRPM5 cells. TRPM5 knock-out (TRPM5^-/-^)^13^ mice were kindly provided by Dr. R. Vennekens, University of Louvain, Belgium.

### iDISCO tissue clearing and immunofluorescence

Adult M5-GFP animals were deeply anesthetized with a mix of ketamine/xylazine and transcardially perfused with PBS followed by 4% ice-cold PFA. Brains were removed and postfixed in 4% PFA on ice for three hours. 250 µm thick sections of the median eminence were cut on a vibratome (Leica). Sections were dehydrated in increasing methanol concentrations, incubated in 66% dichloromethane (DCM)/33% methanol and bleached with 5% H_2_O_2_ in methanol. Sections were then rehydrated in decreasing methanol concentrations and incubated in blocking solution (0.2% gelatin, 0.5% TX-100 in PBS) for three days at RT followed by staining with primary antisera for 1 week at 37 °C diluted in blocking solution. Primary antisera used were rabbit anti-GFP (1:10000, Invitrogen #A-6455, AB_221570), goat anti-CD31 (1:5000, R&D Systems, #AF3628, AB_2161028), chicken anti-vimentin (1:2500, GeneTex, GTX30668, AB_626086) and chicken anti-mouse PV1 (1:5000, directed and affinity-purified against the C-t sequence (CKK)-LPVVNPAAQPSG, custom-prepared by Caslo, Kongens Lyngby, Denmark; labeling identical to that given by the rat monoclonal clone MECA-32 in Ciofi et al.^72^, AB_2892196). Thereafter, incubation in secondary antisera diluted in blocking solution was for 4 days at 37 °C. Secondary antisera (all from Jackson Immunoresearch) used were donkey anti-rabbit Cy5 (1:500, # 711-175-152), donkey anti-goat Cy2 (1:500, #705-225-147), donkey anti-goat Cy3 (1:500, #705-165-147), donkey anti-chicken 488 (1:500, #703-545-155) and donkey anti-chicken Cy3 (1:500, #703-165-155). After staining, sections were dehydrated and incubated in 66% DCM/33% methanol overnight at 4 °C. On the next day, sections were washed once in DCM and then cleared in dibenzylether (DBE). Cleared sections were mounted on a glass slide with DBE and imaged with a confocal or SIM microscope (both from Zeiss). Image stacks were analyzed and videos were prepared with the Imaris software package (Bitplane).

### AAV vector production

An AAV expressing mCherry under the control of the CAGs promoter was generated in a combined 1 and 2 serotype using the triple transfection helper-free method. In brief, HEK293T cells in culture were transfected with 3 plasmids in a 1:(0.5:0.5):1 ratio; the first containing essential viral genes such as E2 and E4 (pAdDeltaF6; Addgene plasmid # 112867), the second which determines the AAV serotype was in this case equimolar amounts of both serotype 1 (AAV2/1; Addgene plasmid # 112862) and serotype 2 (AAV2/2; Addgene plasmid # 104963) plasmids to generate AAV particles of a mixed 1 and 2 serotype and the third which dictates the packaged contents of the virus particles and contained mCherry under control of the CAGs promoter both flanked by two ITR sites (Addgene plasmid # 91947). Transfection was undertaken when the cells reached 60-70% confluence using a 4:1 (v:w) ratio of Polyethylenimine (PEI) to plasmid DNA. 60 – 72 hours after transfection, both supernatant and cells were processed to recover the virus. Viral titer was measured by qPCR analysis with primers specific to the ITR region of the packaging plasmid (fwd ITR primer: 5’-GGAACCCCTAGTGATGGAGTT, rev ITR primer: 5’-CGGCCTCAGTGAGCGA). We named this mixed serotype virus AAV2/1 + 2-CAGS-mCherry.

### M5 tanycyte enrichment, RNA-seq library building and sequencing

M5-GFP and M5-DREADD-GFP mice were i.c.v. injected with 2 μl AAV2/1 + 2-CAGS-mCherry virus three weeks before cell sorting. The basal hypothalamus from adult male M5-GFP mice, which either underwent 14 hours of fasting (8 mice) or normal diet feeding (6 mice) or from 6 adult male M5-DREADD-GFP mice (M5-GFP mice were used as control (6 mice), animals were injected with 2 µg CNO i.c.v. and sacrificed after 3 hours) were dissected and pooled from 2 mice. Dissociation was performed using a Papain dissociation system (Worthington) as described previously^73^. The dissociated cells were then sorted using FACS (Sony SH800, Software version 2.1.5, Supplementary Fig. 12). Cells were sorted by fluorescence (endogenously expressed GFP and virally expressed mCherry) with excitation at 488 nm and 561 nm and emission detected at FL2 (525/50 nm) and FL3 (600/60 nm). Approximately 1000 sorted M5 tanycytes for each condition were used to build RNA-seq libraries using the Smart-seq2 method. The sequencing and analyses were performed by Novogene Europe. Briefly, total RNA was purified from sorted cells using the RNeasy plus Micro kit (Qiagen, Hilden, Germany). Total RNA was then amplified using the SMART-Seq v4 Ultra Low Input RNA kit for Sequencing (Takara Bio USA, Mountain View, USA) synthesizing double stranded cDNA (ds-cDNA). The ds-cDNA was then purified with AMPure XP beads and quantified with Qubit (Life Technologies). The library preparation was performed using the NEB Next Ultra RNA Library Prep kit (New England Biolabs, Ipswich, USA) following the manufacturer’s recommendations. Libraries were sequenced on an Illumina NovaSeq 6000 S4 flowcell with PE150. Raw reads were subjected to quality control and then trimmed for library adapters and low-quality tails. Trimmed reads were mapped to the mouse reference genome (mm10) using STAR (version v2.6.1d)^74^. The quantification of read numbers mapped of each gene was performed by FeatureCounts^75^, and then differential expression analysis was performed using DESeq2^76^ with screening analysis threshold set to p-value<0.05 and |log2(FoldChange)|>1. The clusterProfiler R package^77^ was used to perform GO and KEGG pathway enrichment analyses.

### Acute brain slice preparation and calcium imaging

M5-GCaMP mice were sacrificed by rapid cervical dislocation. The brain was extracted from the skull and transferred to ice-cold cutting solution (containing in mM: NaCl 87, KCl 3, NaH_2_PO_4_ 1.25, NaHCO_3_ 25, glucose 10, sucrose 75, MgCl_2_ 1, CaCl_2_ 0.5). During sectioning, the brain was immersed in the ice-cold cutting solution and continuously aerated with 95% O_2_/5% CO_2_. 200 μm thick coronal sections were obtained using a Leica VT1200S vibratome. The slices were incubated for at least 30 min in artificial cerebrospinal fluid (ACSF, containing in mM: NaCl 120, KCl 3, NaH_2_PO_4_ 1.25, NaHCO_3_ 25, glucose 10, MgCl_2_ 1, CaCl_2_ 2) at 35-37 °C before calcium imaging.

For the cell loading experiments, coronal sections (200 μm) were loaded with a fluorescent Ca^2+^ indicator Cal520 (10 μΜ) during 1 h in aerated ACSF (95% O_2_/5% CO_2_) at RT. Following this, the sections were kept in aerated ACSF for at least 30 minutes before imaging. Imaging of the calcium signals in M5 tanycytes was performed with a Zeiss LSM 710 confocal imaging system operated by the ZEN software (Carl Zeiss ZEN 2012 (black) 64 bits, Version 14). To hold the brain slice, a RC-26G Open Diamond Bath Imaging Chamber (Warner Instruments) was routinely used. For the chamber floor, glass coverslips (22×40 mm, CS-22/40, Warner Instruments) were prepared as follows. The coverslips were soaked in nitric acid (70%, Sigma Aldrich) overnight and then washed with distilled water until reaching pH ~7.0. The coverslips were attached to the chamber with vacuum grease silicone (Beckman Coulter, Cat. No. 335148), the brain slice was then transferred into the chamber and attached to the floor by short solution removal followed by replenishment of the ACSF in the chamber. The solution flow rate through the chamber was kept at 2 ml/min using a custom-made tube perfusion system. During imaging, all bath applied solutions were aerated (95% O_2_/5% CO_2_). In the experiments with local application, solutions were applied via a patch pipette. For this, glass pipettes pulled from borosilicate glass tubes (GB150T-8P, SCIENCE PRODUCTS GmbH) were routinely used. The opening of a patch pipette had electrical resistance of 3-5 MOhm in ACSF. Imaging was done using a 20x water immersion objective (W Plan-Apochromat 20x/1,0 DIC VIS-IR, Carl Zeiss), which allowed observation of the whole median eminence in coronal sections prepared from adult mice. The frequency of frame collection was set to 2 Hz. Calcium imaging corrections and statistical analyses were done with MATLAB_2021b (Mathworks, Natick, MA, USA). Heatmaps for the fluorescence responses throughout the ME were made using MATLAB as follows. On a representative image of a coronal section, a line around the third ventricle border was marked touching tanycytic cell bodies. Pixel intensities above a fluorescent threshold and outside the third ventricle line (i.e., inside the brain slice) were taken for the further analysis. All intensities, each calculated as ΔF/F0, from pixels which were equidistant to the third ventricle line were summed up and normalized by the number of pixels, giving a fluorescence of the ME at a particular distance from the third ventricle. This was done for every time frame giving one vertical line for each time point. Then all the vertical lines were organized in chronological order from left to right. The values in the heatmap were color coded as indicated in the corresponding figure and figure legend.

### Surgery and tissue preparation

Twelve to sixteen-week-old M5-GFP or M5-DREADD mice were anesthetized with 5% isoflurane mixed with oxygen and then transferred to a stereotaxic injection apparatus (Stoelting Co.). Anesthetized mice were maintained under 2% isoflurane. The scalp was shaved and opened with a scalpel along the midline to expose the skull. The tissue was cleaned with 3% H_2_O_2_. According to the lateral ventricle coordinates given in the Franklin and Paxinos Atlas^78^, one hole was drilled into the cranium using a dentist drill. With a Hamilton microliter syringe, 2 μl of 3 mM stevioside, 2 µg leptin, 2 μg CNO in saline or 2 μl saline as control was injected into the lateral ventricle (0.46 mm posterior, 2.50 mm ventral and 1.00 mm lateral to bregma) or the third ventricle (1.79 mm posterior, 5.80 mm ventral to bregma). Mice were then transcardially perfused with PBS followed by 4% paraformaldehyde in PBS. Brains were removed, postfixed for 2 hours in 4% paraformaldehyde in PBS, and then placed in a 30% sucrose solution. Brains were frozen in tissue-freezing medium (Leica, Nussloch, Germany) in a dry ice/ethanol slurry. 14 μm thick coronal sections were cut using a cryostat (Leica) and stored at −80 °C until use.

### Quantification of serum hormone levels

Twelve to sixteen-week-old wild type or M5-DREADD mice were anesthetized with isoflurane mixed with oxygen and injected with 2 μg CNO in saline into the third ventricle. Mice were removed from stereotaxic injection apparatus and cheek blood was taken at various time points. The blood was allowed to clot before centrifugation at 1000 g for 10 min to collect serum. Serum hormone levels were measured via Luminex MAGPIX System. Insulin and leptin levels were measured via mouse metabolic magnetic bead panel (Cat. # MMHMAG-44K). Adrenocorticotropic hormone (ACTH), follicle-stimulating hormone (FSH), prolactin (PRL), thyroid stimulating hormone (TSH) and growth hormone (GH) level were measured via mouse pituitary magnetic bead panel (Cat. # MPTMAG-49K). Briefly, 200 μl assay buffer was added into each well of a 96-well plate and shaken for 10 min. Assay buffer was removed and 10 μl of either matrix solution, assay buffer, standard or control and samples was added into appropriate wells. A total of 25 μl beads were added to each well and this was incubated overnight at 4 °C with shaking. Well content was removed and beads washed with wash buffer. A total of 50 μl detection antibody was added to each well and incubated 30 min at room temperature. 50 μl streptavidin-phycoerythrin was added to each well and incubated for 30 min. Well contents were removed and beads were again washed with wash buffer. 100 μl drive fluid was added to each well and the plate was run on MAGPIX using xPONENT software (version 4.2). Data were analyzed with Prism 5.

### Surgery and metabolic phenotyping

Five-week-old M5-GFP-iDTR, M5-iDTR mice, or WT-iDTR mice were anesthetized then transferred to a stereotaxic injection apparatus as described above. After exposure of the skull, two holes were drilled into the cranium and 0.5 ng diphtheria toxin were injected into both sides of the third ventricle (1.8 mm posterior, 5.8 mm ventral, and 0.25 mm lateral to bregma). Mice were allowed to recover 7 days after surgery. Metabolic phenotyping started 14 days after the surgery. For metabolic phenotyping, mice were kept at an ambient temperature set to 22 ± 2 °C with a constant humidity (45–65%) and a 12 h/12 h light/dark cycle. Mice had free access to water and were fed ad libitum with a high fat diet (58% kcal fat; Research Diets, New Brunswick, NJ, USA; # D12331) since week 7. Intraperitoneal glucose tolerance (ipGTT) was assessed in 30-week-old mice after 5 h fasting and after stimulation with 1.5 g glucose per kg body weight. Body composition (fat and lean tissue mass) was analyzed in 33-week-old mice using a magnetic resonance whole-body composition analyzer (EchoMRI, Houston, TX). Intraperitoneal insulin tolerance (ipITT) was assessed in 48-week-old mice after 3 h fasting with stimulation of 0.75 units insulin (Recombinant Insulin Human, Novo Nordisk) per kg body weight. Plasma insulin and HOMA-IR were evaluated/calculated post ipITT. Energy expenditure, substrate utilization (respiratory exchange ratio, RER) and home-cage activity were assessed in 33-week-old mice using a climate-controlled indirect calorimetric system (TSE System, Bad Homburg, Germany). After acclimatization for 24 h, levels of O_2_ and CO_2_ were measured every 10 min for 4–5 days. Intraperitoneal glucose tolerance of WT-DREADD mice and WT mice not containing the DREADD allele (12–16 week old) were performed 3 weeks after 2 μl AAV-DIO2-Cre virus injection into the cerebral ventricle. Mice were fasted 12 h and injected with a mixture of 1 mg CNO-HCl and 2 g glucose per kg body weight.

### Immunohistochemistry

Sections were washed with PBS and blocked in 10% donkey serum/3% BSA/0.3% Triton X-100 in PBS for 1 h at room temperature. Sections were incubated with primary antisera overnight at 4 °C followed by secondary antisera at room temperature for 2 hours. Antisera used were as follows: rabbit anti-c-Fos (1:500, Cell Signaling Technology, #2250, AB_2247211), chicken anti-GFP (1:1000, ThermoFisher, #A10262, AB_2534023), chicken anti-HA tag (1:1000, ThermoFisher, #PA5-33243, AB_2550658), chicken anti-vimentin (1:500, GeneTex, GTX30668, AB_626086), goat anti-human HB-EGF (1:1000, R&D Systems, #AF-259-NA), rabbit anti DsRed (1:1000, TaKaRa, #632496), goat anti-chicken Alexa 488 (1:500, Invitrogen, #A11039), goat anti-rabbit cy3 (1:500, Invitrogen, #A10520), donkey anti-goat cy3 (1:1000, Jackson Immuno Research, #705-165-147) and donkey anti-chicken 488 (1:500, Jackson Immuno Research, #703-545-155). For p-STAT5 staining, sections were washed with TBS, antigen retrieval was performed at 95 °C in 0.01 M Tris for 2 min, then blocked in 0.25% BSA/0.3% Triton X-100 in TBS for 10 min at room temperature. Sections were washed with TBS and incubated with rabbit anti-pSTAT5 (1:600, Cell Signaling Technology, #9359, AB_823649) overnight at 4 °C followed by goat anti-rabbit Cy3 (1:500, Jackson Immunoresearch, #711-165-152) at room temperature for 2 h. Cell nuclei were counterstained with bisbenzimide (Sigma). Images were captured using a Zeiss Axio Imager 2 microscope. Counting procedures for p-STAT5 and c-Fos are described below.

### Evans blue injection

WT-DTA and M5-DTA mice were subjected to tail vein injection of 50 μl 1% Evans blue dissolved in 0.9% saline. Mice were sacrificed 20 min later by decapitation. The brains of the injected mice were removed and quickly frozen in O.C.T. (Leica). 14 μm sections were obtained on a cryostat and stored at −80 °C until analysis. Evans blue signal was observed by fluorescence microscopy. Area covered by Evans blue was measured with ZenBlue software.

### Quantification

p-STAT5 and c-Fos positive cells were manually counted in every tenth section in 14 μm coronal sections obtained serially across the median eminence. Counted numbers for each section were added together and averaged to give an average number of c-fos or p-STAT5 positive tanycytes per section for each mouse.

Areas positive for DTR immunosignal within the pars tuberalis and choroid plexus were calculated for every tenth section in 14 μm coronal sections obtained serially along the median eminence and ventricle using Zen 2.3 software (Zeiss).

### Statistical methods

Statistical analyses of the calcium imaging data were performed using MatLab2021b (Mathworks, Natick, MA, USA). The data were collected and analyzed offline. Single measurements of individual tanycytes were baseline-corrected, grouped and are presented as mean and median. Comparisons between groups were done with N-way ANOVA tests, followed by Tukey’s or Bonferronis post-hoc tests. The effects of substances on the tanycytes were measured as calcium elevations, presented with mean and median, and compared via paired two-sided t-tests. Statistical differences were considered significant at *, *p* < 0.05; **, *p* < 0.01; ***, *p* < 0.001; ****, *p* < 0.0001. Data of energy expenditure were analyzed using ANCOVA with body weight as covariate as previously described^79,80^.

### Reporting summary

Further information on research design is available in the Nature Portfolio Reporting Summary linked to this article.

## Supplementary information

Supplementary Information

Description of Additional Supplementary Files

Supplementary Movie 1

Supplementary Movie 2

Supplementary Movie 3

Reporting Summary

## Data Availability

Raw sequencing data have been deposited on GEO under accession code GSE226066. The reference genome used for comparison was the mouse reference genome (mm10, GEO accession: GSM6042958). All other data generated or analyzed during this study are included in this published article (and its supplementary information files). Source data are provided with this paper.

## Source data

Source Data
