# Supplementary material for: Bitter taste cells in the ventricular walls of the murine brain regulate glucose homeostasis
Source: Nat Commun. 2023 Mar 22;14:1588. doi: 10.1038/s41467-023-37099-3 (PMC10033832; doi:10.1038/s41467-023-37099-3)
Supplement: Supplementary file 1 — Supplementary Information [file 41467_2023_37099_MOESM1_ESM.pdf]

SUPPLEMENTARY MATERIAL

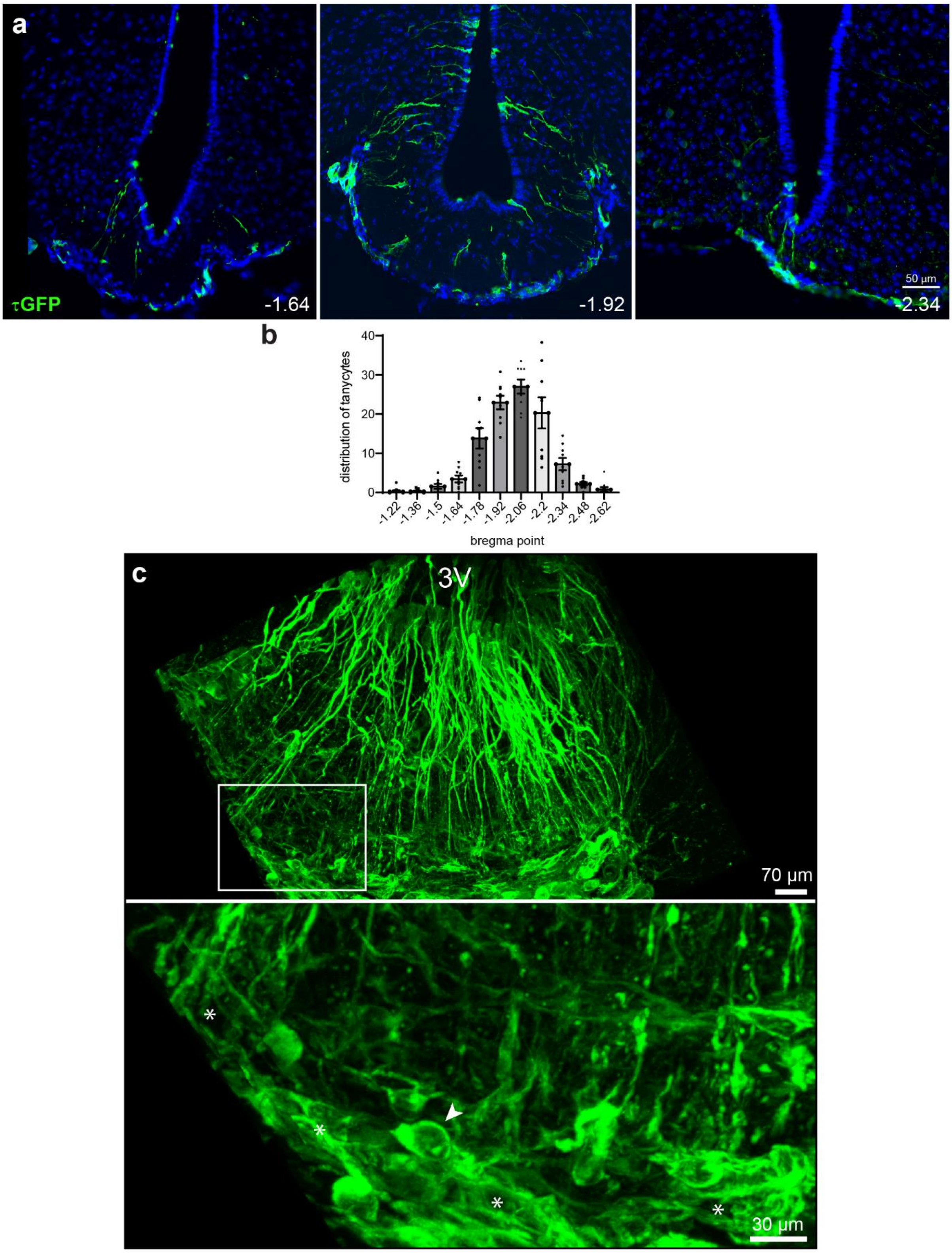

Supplementary Fig. 1: a M5 tanycytes (green, n = 9) are in the walls of the third ventricle. **b** Distribution of M5 tanycytes at the level of the third ventricle (n = 9) are displayed according to the indicated bregma coordinates. **c** iDISCO cleared ME (n = 3) shows TRPM5 tanycytic processes

(asterisks) and stained cells in the *pars tuberalis* (arrowheads). 3V: third ventricle. Error bars represent the standard error of the mean. Source data are provided as a Source Data file.

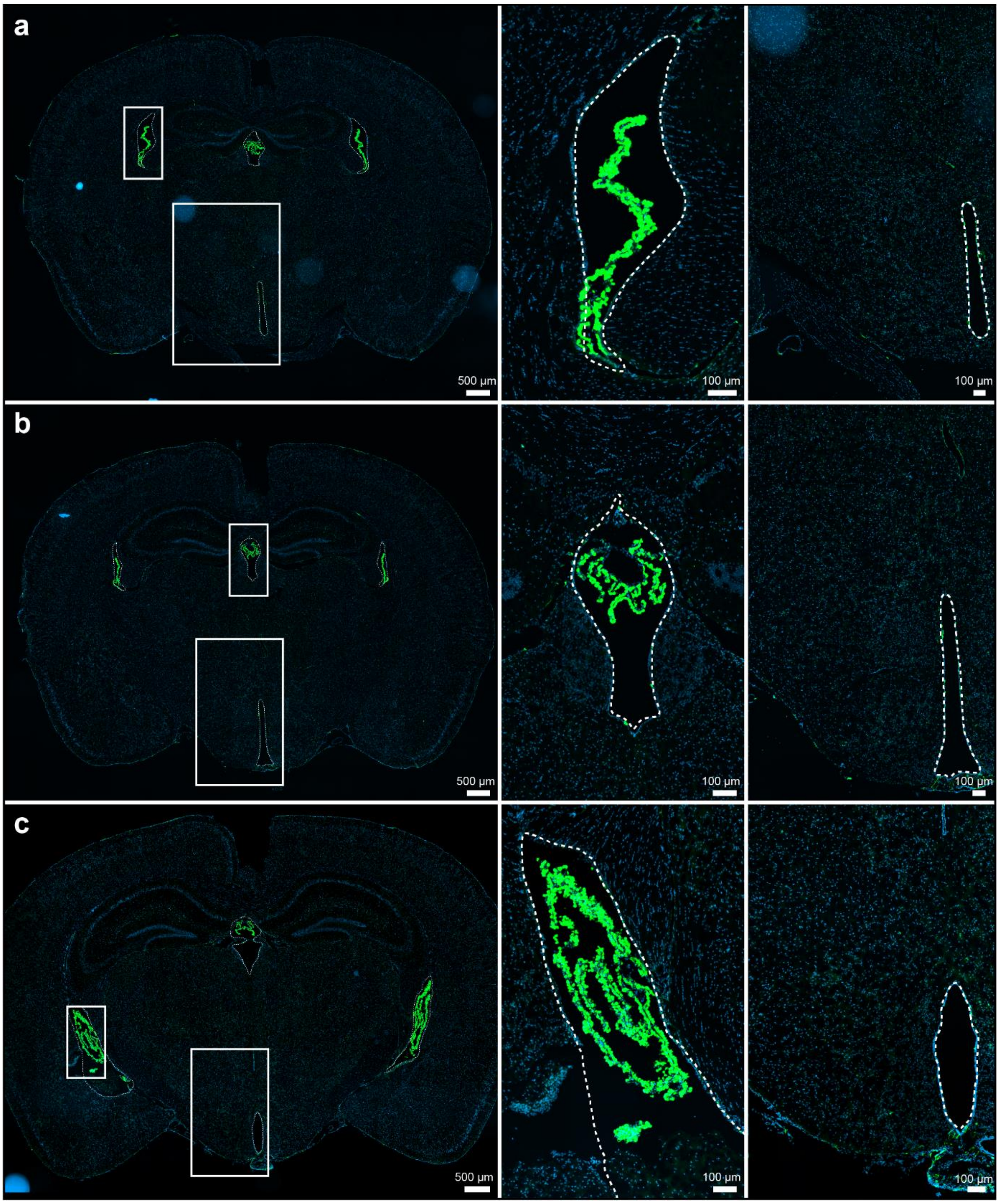

Supplementary Fig. 2: TRPM5 cells (green, n = 3) are found in the choroid plexus. **a-c** Overview of TRPM5 cells in the hypothalamus and choroid plexus in 14 µm cryosections from rostral (a) to caudal (c). TRPM5 cells were found in the choroid plexus and in tanycytes at the base of the third ventricle. Scalebars: 500 µm (overview), 100 µm (insets).

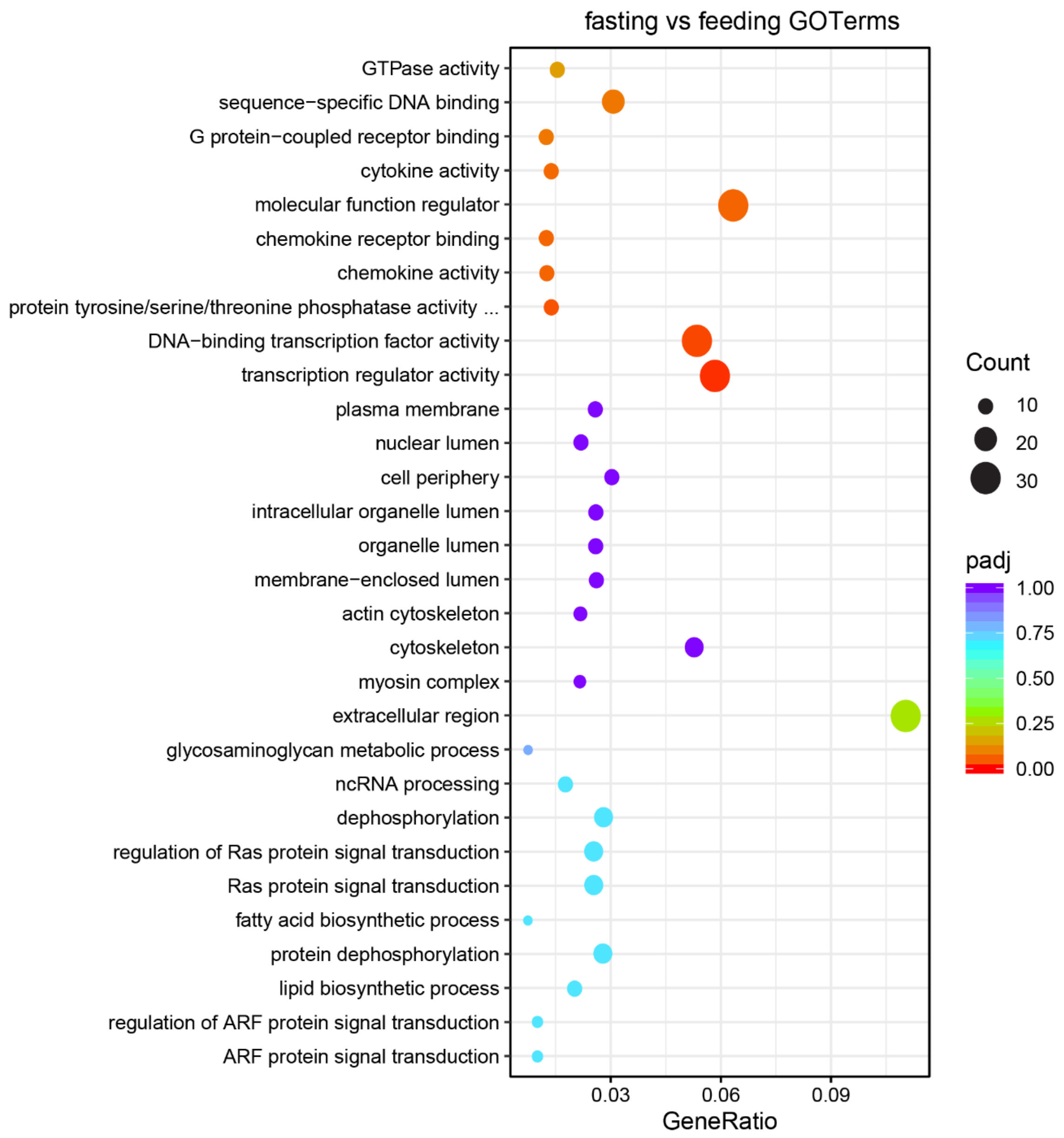

Supplementary Fig. 3: Scatter Plot of GO terms enriched from all the differentially expressed genes. The color and size of the dots are scaled with respect to padj value and the number of the differentially expressed genes, respectively. Padj were obtained via hypergeometric test, and FDR correction was done using the Benjamini and Hochberg method.

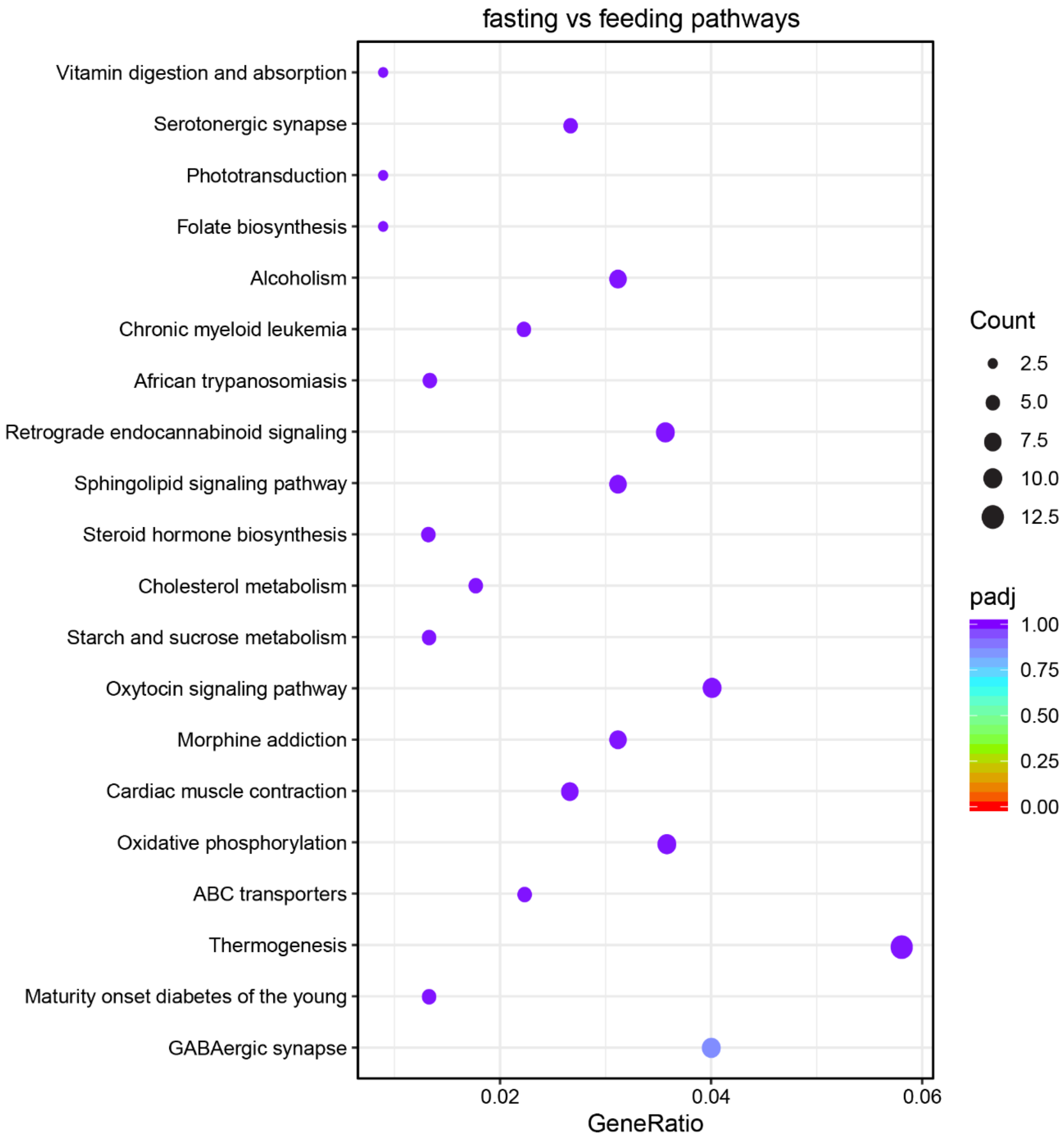

Supplementary Fig. 4: Scatter Plot of KEGG pathway enriched from all the differentially expressed genes. The color and size of the dots are scaled with respect to padj value and the number of the differentially expressed genes, respectively. Padj were obtained via hypergeometric test, and FDR correction was done using the Benjamini and Hochberg method.

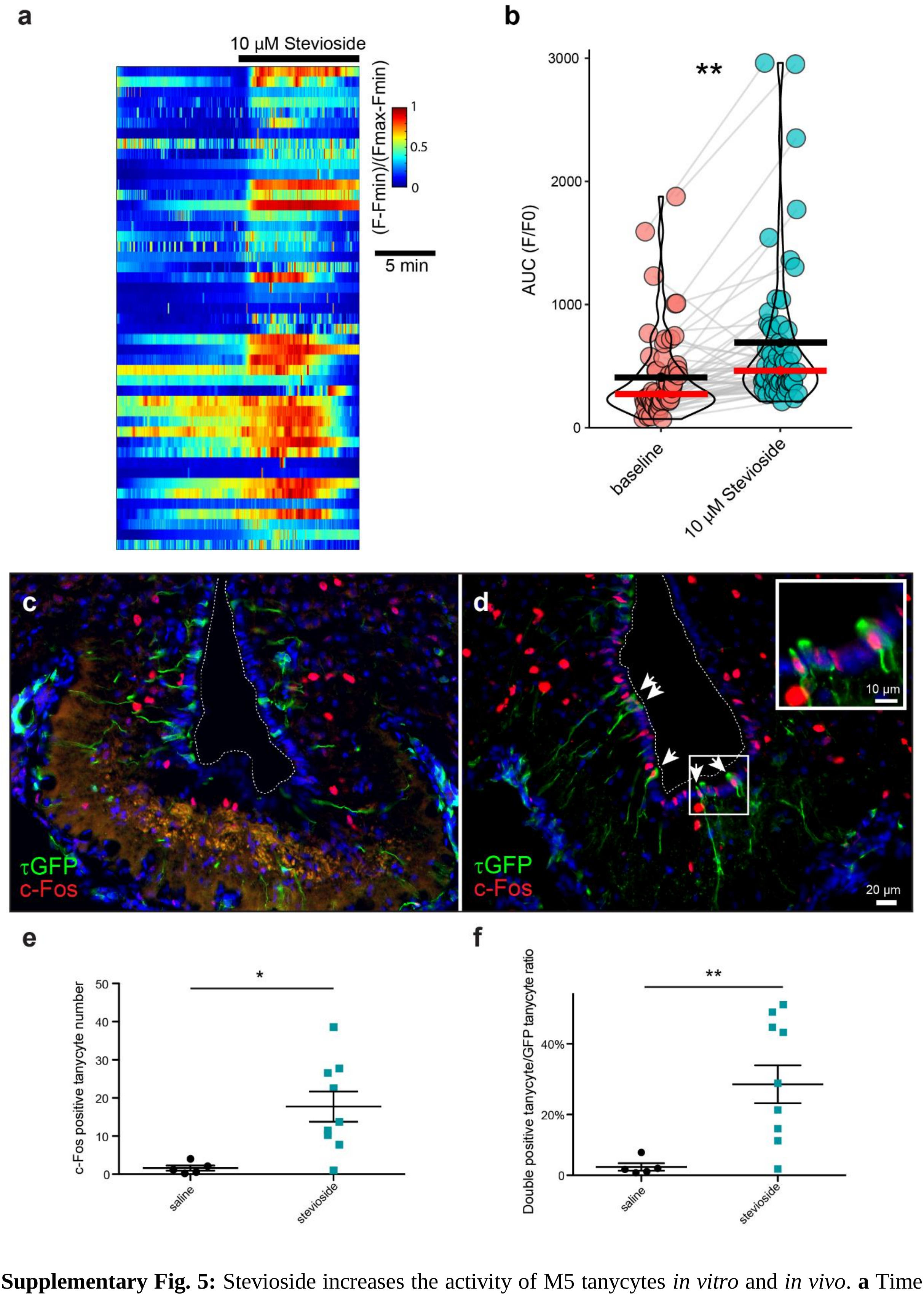

Supplementary Fig. 5: Stevioside increases the activity of M5 tanycytes *in vitro* and *in vivo*. **a** Time course heatmap of the GCaMP3 normalized fluorescent changes after 10 µM stevioside application 10 min baseline vs 10 min application. **b** Area under the curve (AUC) of the normalized intensities of the M5 tanycytes. Measurements of every cell before and after stevioside application. Red lines are median,

black lines are means for each group. Means were compared via two-sided t-test (p=0.0033, for n=3). **c**, **d** Stevioside activates M5 tanycytes *in vivo*. **c** M5-GFP mice were intracerebroventricularly injected with 2 µl saline and perfused 1 hour later (n=5). **d** M5-GFP mice were intracerebroventricularly injected with 2 µl of 3 mM stevioside and perfused 1 hour later (n=9). Note the colocalization of the GFP and c-Fos signals. Scalebars: 20 µm (overview), 10 µm (inset). (**e**, **f**) Quantification of c-Fos positive tanycytes (*p*=0.0117) and ratio of tanycytes double positive for both c-Fos and GFP (*p*=0.0079) Data analyzed with 2-tailed, 2-sided t-test. Error bars represent the standard error of the mean. Source data are provided as a Source Data file.

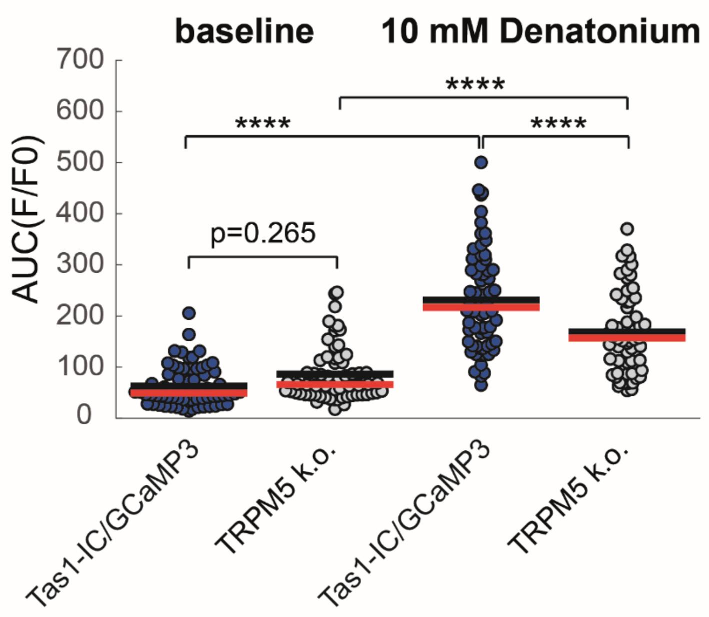

Supplementary Fig. 6: Tanycytic responses to denatonium. Area under the curve (AUC) for Ca^{2+} transients observed from cell bodies of the control mice (n=6) versus TRPM5 knock-out mice (n=4). Statistical comparison between these genotypes of the basal cellular activities (baseline) versus 10 mM denatonium application was done with two-way ANOVA (between baseline measurements p=0.265, between other groups $p < 4{,}25*10^{-6}$). Source data are provided as a Source Data file.

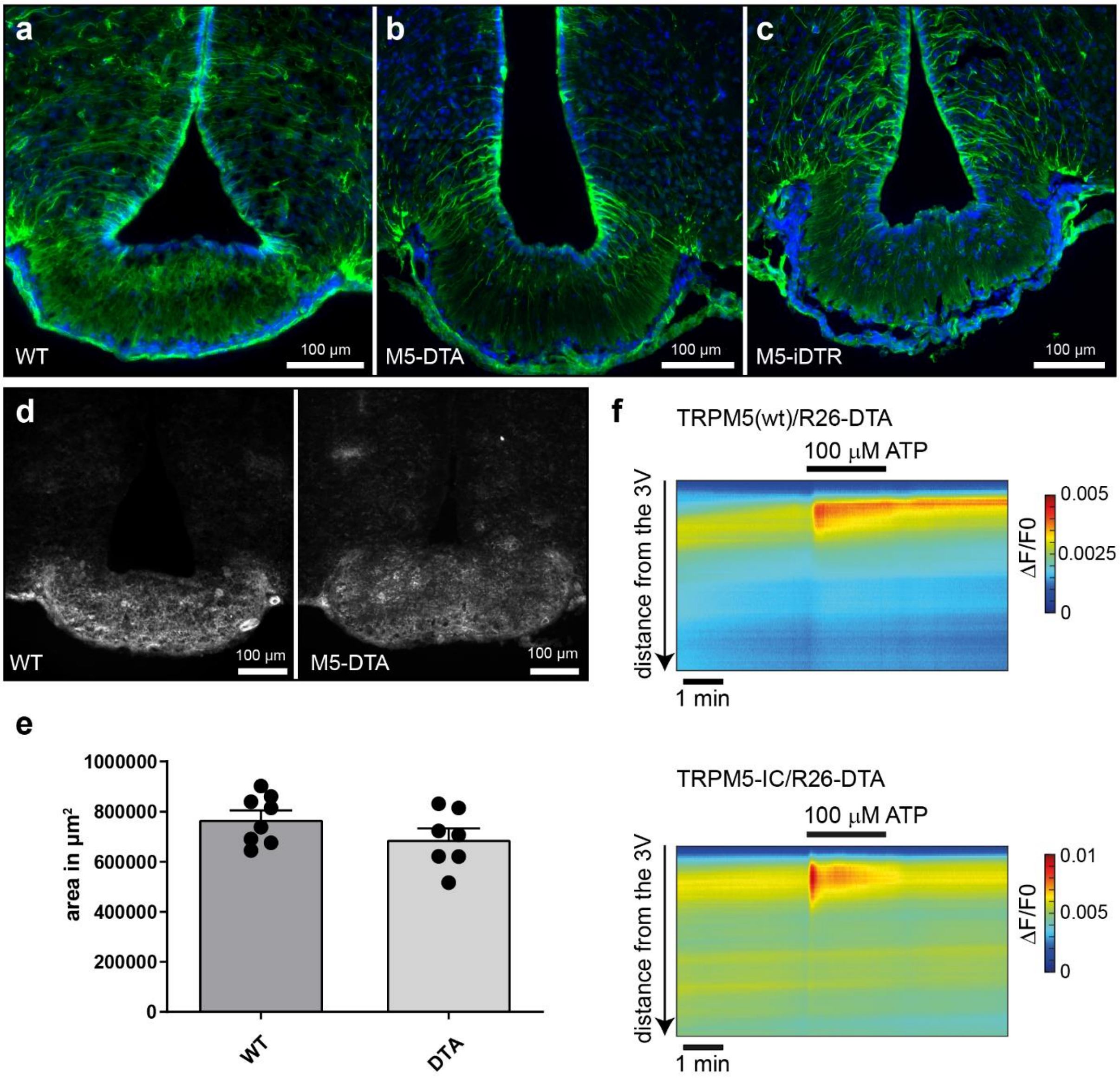

Supplementary Fig. 7: Demonstration of intact barrier in M5 ablated animals. **a-c** Vimentin staining (green) in WT (n=3), M5-DTA (n=3) and DT-injected M5-iDTR animals (n=3). No obvious difference between the genotypes was observed. **d, e** Evans Blue tail vain injection in WT and M5-DTA animals demonstrates no difference in barrier function between the genotypes (n=8 for WT animals and n=7 for ablated animals, scale bars: 100 µm, statistical test used: two-tailed unpaired t-test). **f** Representative responses to 100 µM ATP of the ME loaded with Cal520 prepared from WT-DTA (control) and M5-DTA mice, showed as diagrams of fluorescence across the slice vs time (black time bar 1 min). Error bars represent the standard error of the mean. Source data are provided as a Source Data file.

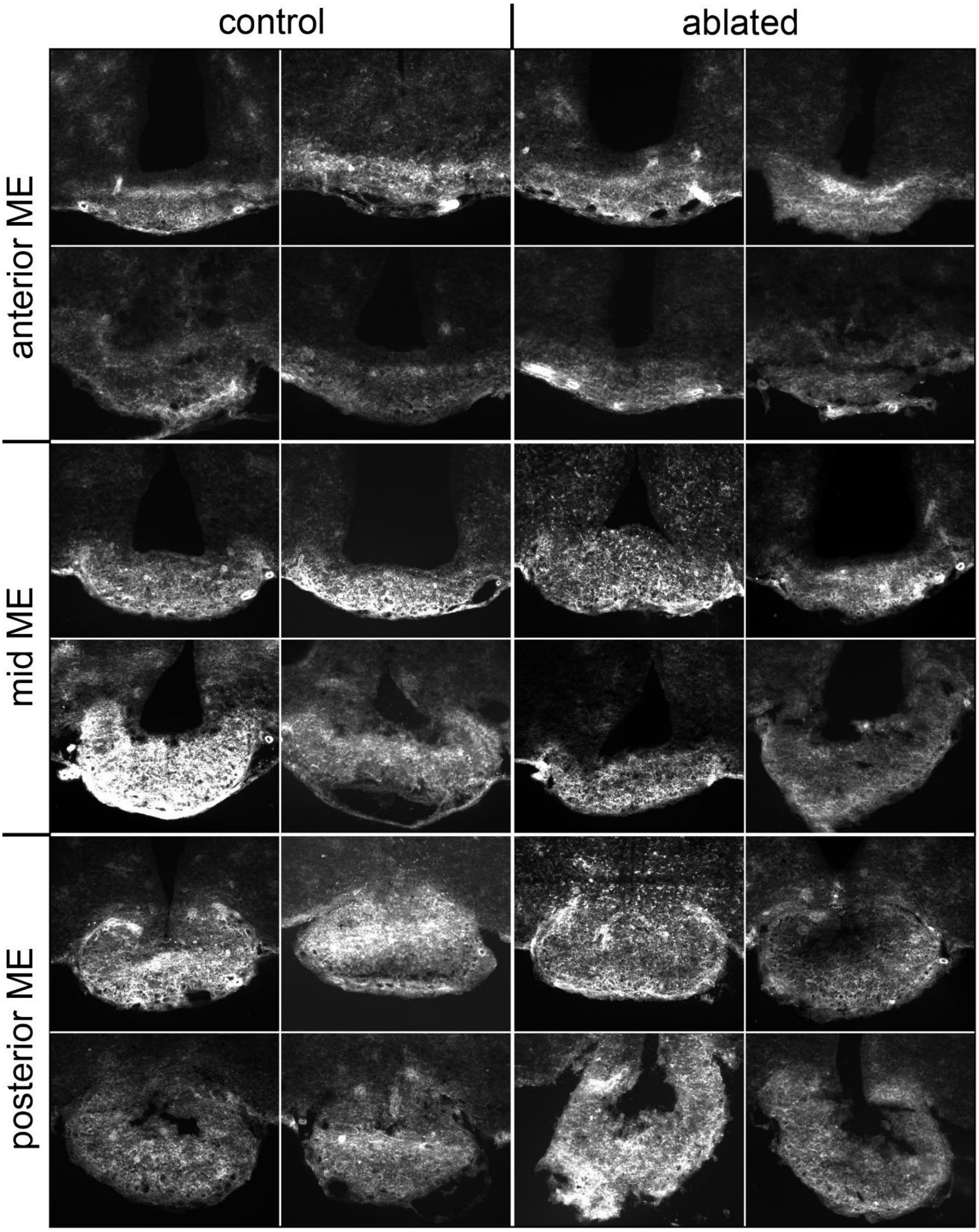

Supplementary Fig. 8: Evans Blue signal in the ME of individual animals in different parts of the ME. Shown is the area covered by Evans Blue in the ME of 12 individual ablated and control animals to illustrate injection dependent variability. Source data are provided as a Source Data file.

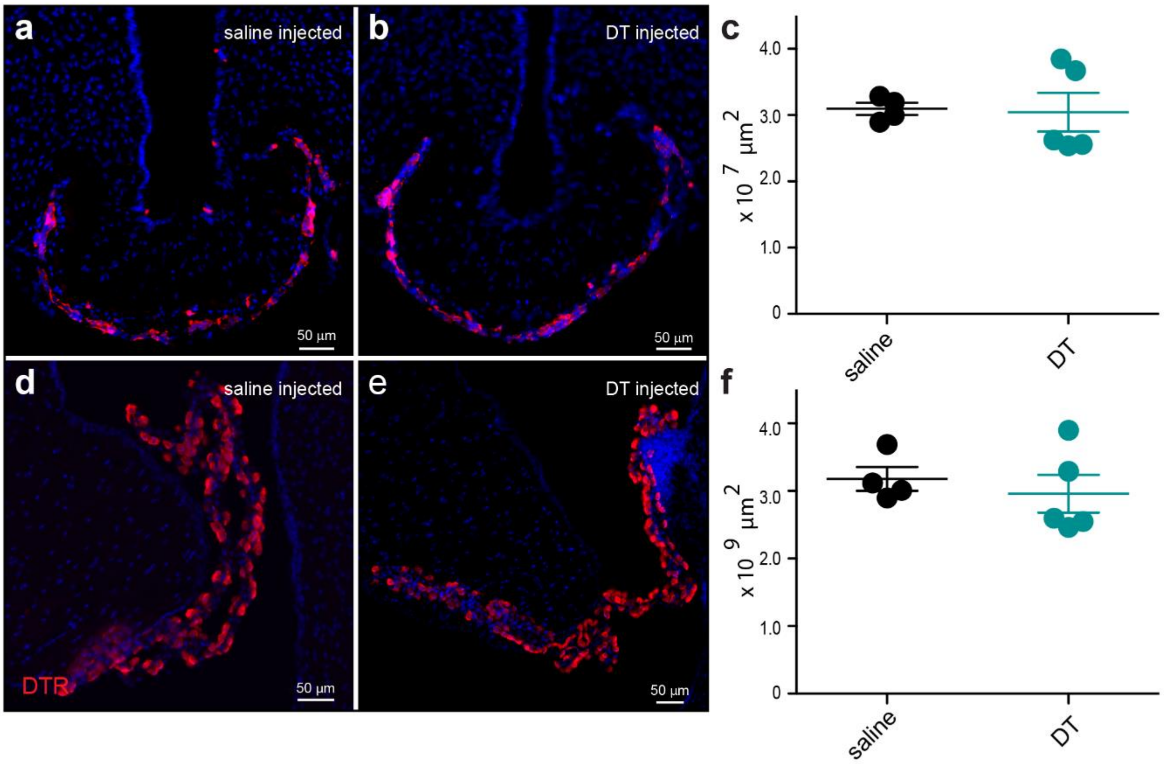

Supplementary Fig. 9: DTR positive area of *pars tuberalis* and choroid plexus after saline (n=4) or DT (n=5) injection into M5-iDTR mice. **a-c** DTR staining of *pars tuberalis* and statistical analysis. **d-f** DTR staining of choroid plexus and statistical analysis. Statistical test used: two-tailed unpaired t-test. Error bars represent the standard error of the mean. Source data are provided as a Source Data file.

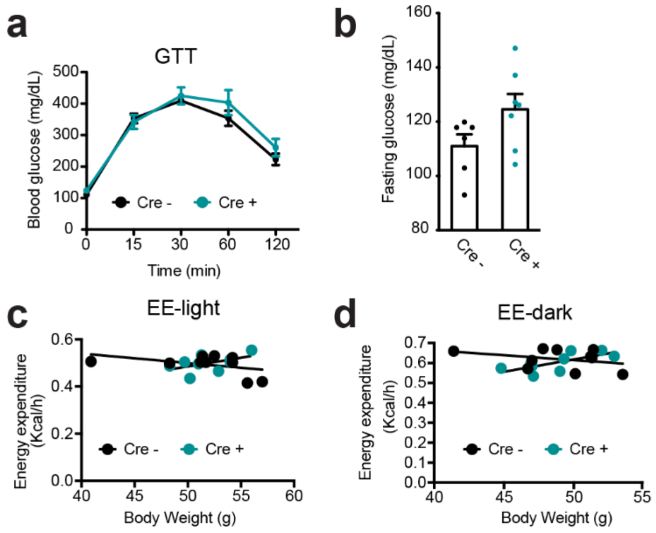

Supplementary Fig. 10: **a**, **b** Glucose tolerance test and baseline glucose level in ablated (n=7) and control animals (n=6) fed with normal chow. Statistical test used: two-tailed unpaired t-test. **c**, **d** Energy expenditure in ablated (n=9) and control animals (n=10) during the light and dark cycle fed with high fat diet. Data in (c and d) were analyzed using ANCOVA with body weight as co-variate. Error bars represent the standard error of the mean. Source data are provided as a Source Data file.

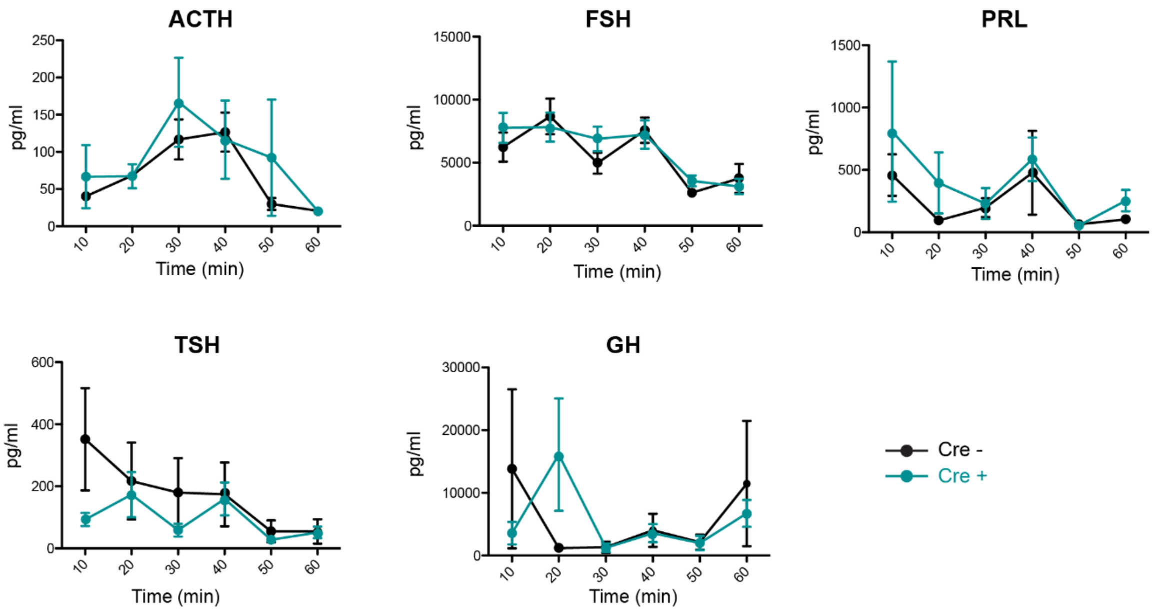

Supplementary Fig. 11: Serum pituitary hormone levels after CNO activation of M5-DREADD tanycytes (blue line for Cre+, black line for Cre-). Blood was taken at different time points from wild type and M5-DREADD mice after 2 μg CNO injection into the third ventricle. ACTH, FSH, PRL, TSH

and GH levels (indicated headlines on the corresponding graphs) were measured via Luminex MAGPIX System (n=4 mice). Statistical test used: two-tailed unpaired t-test. Error bars represent the standard error of the mean. Source data are provided as a Source Data file.

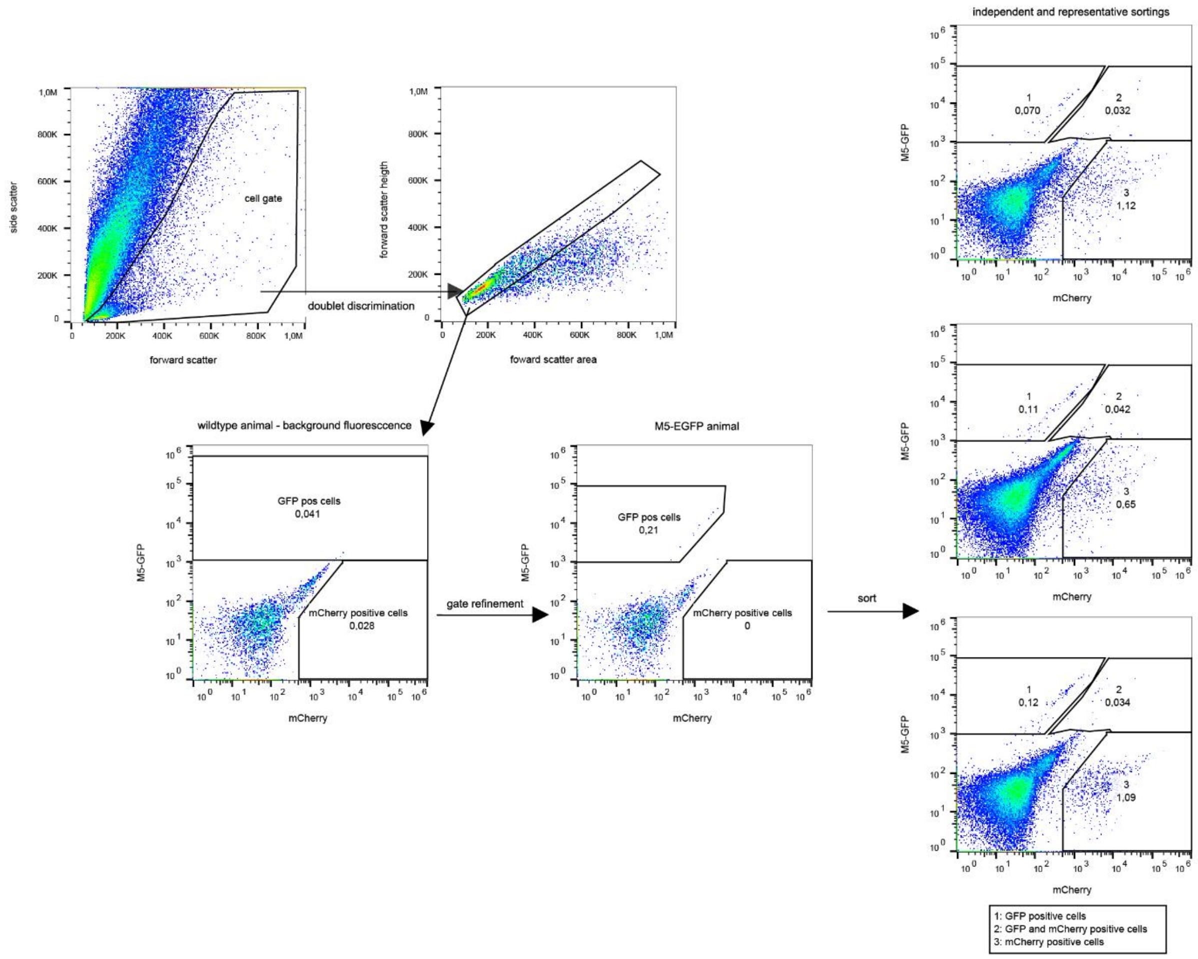

Supplementary Fig. 12: Forward scatter (FSC) and side scatter (SSC) were used firstly to determine the cells and tissue debris, then the area versus the height of FSC was used for doublet discrimination. Samples from wildtype animals and animals with endogenous GFP expression were used to set the initial gates. In the end, cells were sorted by fluorescence (endogenously expressed GFP and mCherry from virus) with excitation at 488 nm and 561 nm, and emission was detected in FL2 (525/50 nm) and FL3 (600/60 nm). The pseudocolor plots of three independent sorts are shown.
