# Supplementary material for: Bitter taste cells in the ventricular walls of the murine brain regulate glucose homeostasis
Source: Nat Commun. 2023 Mar 22;14:1588. doi: 10.1038/s41467-023-37099-3 (PMC10033832; doi:10.1038/s41467-023-37099-3)
Supplement: Supplementary file 2 — Description of Additional Supplementary Files [file 41467_2023_37099_MOESM2_ESM.pdf]

Description of Additional Supplementary Files

File Name: Supplementary Movie 1
Description: Video of an iDISCO-cleared and 3D-reconstructed median eminence (ME) referring to Fig. 1d-g. Video made with Imaris software from a zStack acquired with a confocal microscope. M5 tanycytes (green) form endfeet in close apposition to CD 31-positive blood vessels (red). One tanycyte process branches multiple times into thinner processes the closer they get to the blood vessel. The video highlights these endfeet originating from two tanycytes from different angles. Scalebar is indicated in the bottom left corner

File Name: Supplementary Movie 2
Description: Video of an iDISCO-cleared and 3D-reconstructed median eminence (ME) referring to Fig. 1h-j. Video made with Imaris software from a zStack acquired with a SIM microscope. M5 tanycytes (green) form endfeet in close apposition to PV-1-positive (red) fenestrations. The video highlights one endfoot closely approaching fenestrations with 0.5 µm distance. Please note that tanycyte endfeet are still branching as close as 1 µm to the fenestration. The second part of the video highlights endfeet closely approaching fenestrations from different angles.

File Name: Supplementary Movie 3
Description: Ca2+ wave propagation alongside M5 tanycytes. Confocal imaging of 300 µm thick coronal brain slice with ME from a 12 week old male M5-GCaMP mouse. Recording of spontaneous activity shows bidirectional Ca2+ wave propagation alongside tanycytes, while the increase in green signal intensity indicates corresponding [Ca2+] rise. The video was recorded in grayscale, green color was introduced as a demonstration of GCaMP3 emission wavelength. Frame rate of the video was changed to 16x faster.
