# Supplementary material for: Bitter taste cells in the ventricular walls of the murine brain regulate glucose homeostasis
Source: Nat Commun. 2023 Mar 22;14:1588. doi: 10.1038/s41467-023-37099-3 (PMC10033832; doi:10.1038/s41467-023-37099-3)
Supplement: Supplementary file 6 — Reporting Summary [file 41467_2023_37099_MOESM6_ESM.pdf]

nature portfolio

Corresponding author(s): Professor Ulrich Boehm (ulrich.boehm@uks.eu)

Last updated by author(s): Feb 23, 2023

Reporting Summary

Nature Portfolio wishes to improve the reproducibility of the work that we publish. This form provides structure for consistency and transparency in reporting. For further information on Nature Portfolio policies, see our Editorial Policies and the Editorial Policy Checklist.

Statistics

For all statistical analyses, confirm that the following items are present in the figure legend, table legend, main text, or Methods section.

n/a	Confirmed	
☐	☒	The exact sample size (*n*) for each experimental group/condition, given as a discrete number and unit of measurement
☐	☒	A statement on whether measurements were taken from distinct samples or whether the same sample was measured repeatedly
☐	☒	The statistical test(s) used AND whether they are one- or two-sided<br>*Only common tests should be described solely by name; describe more complex techniques in the Methods section.*
☐	☒	A description of all covariates tested
☐	☒	A description of any assumptions or corrections, such as tests of normality and adjustment for multiple comparisons
☐	☒	A full description of the statistical parameters including central tendency (e.g. means) or other basic estimates (e.g. regression coefficient) AND variation (e.g. standard deviation) or associated estimates of uncertainty (e.g. confidence intervals)
☐	☒	For null hypothesis testing, the test statistic (e.g. *F*, *t*, *r*) with confidence intervals, effect sizes, degrees of freedom and *P* value noted<br>*Give P values as exact values whenever suitable.*
☒	☐	For Bayesian analysis, information on the choice of priors and Markov chain Monte Carlo settings
☐	☒	For hierarchical and complex designs, identification of the appropriate level for tests and full reporting of outcomes
☒	☐	Estimates of effect sizes (e.g. Cohen's *d*, Pearson's *r*), indicating how they were calculated

Our web collection on statistics for biologists contains articles on many of the points above.

Software and code

Policy information about availability of computer code

Data collection	Data for imaging was collected with Zen((Carl Zeiss ZEN 2012 (black) 64 bits, Version 14)). For Luminex test, xPONENT Software (version 4.2) was used. For flow cytometry test, SH800S Cell Sorter Software (version 2.1.5) was used.
Data analysis	Data analysis was done with Imaris(Bitplane, version 9.6), Prism (version 5.0b), ZenBlue (Zeiss, version 3.1) and Matlab(Mathworks, Software version (R2021b Update 1 (9.11.0.1809720)). Analysis of the data and building figures was done with standard in-built functions in Matlab and Prism.<br>For RNA-seq:Raw reads were subjected to quality control and then trimmed for library adapters and low-quality tails. Trimmed reads were mapped to the mouse reference genome (mm10, GEO accession: GSM6042958) using STAR (version v2.6.1d). The quantification of read numbers mapped of each gene was performed by FeatureCounts (version 1.5.0-p3), and then differential expression analysis was performed using DESeq2 (version 1.20.0). Genes with padj <0.05 and FPKM >1 in at least one condition were considered to be differentially expressed. The clusterProfiler R package (version 3.8.1) was used to perform GO and KEGG pathway enrichment analyses.

For manuscripts utilizing custom algorithms or software that are central to the research but not yet described in published literature, software must be made available to editors and reviewers. We strongly encourage code deposition in a community repository (e.g. GitHub). See the Nature Portfolio guidelines for submitting code & software for further information.

March 2021

Data

Policy information about availability of data

All manuscripts must include a data availability statement. This statement should provide the following information, where applicable:

- Accession codes, unique identifiers, or web links for publicly available datasets
- A description of any restrictions on data availability
- For clinical datasets or third party data, please ensure that the statement adheres to our policy

Raw sequencing data have been deposited on GEO under accession code GSE226066 [https://www.ncbi.nlm.nih.gov/geo/query/acc.cgi?acc= GSE226066]. Mouse reference genome (mm10, GEO accession: GSM6042958).

Human research participants

Policy information about studies involving human research participants and Sex and Gender in Research.

Reporting on sex and gender	*Use the terms sex (biological attribute) and gender (shaped by social and cultural circumstances) carefully in order to avoid confusing both terms. Indicate if findings apply to only one sex or gender; describe whether sex and gender were considered in study design whether sex and/or gender was determined based on self-reporting or assigned and methods used. Provide in the source data disaggregated sex and gender data where this information has been collected, and consent has been obtained for sharing of individual-level data; provide overall numbers in this Reporting Summary. Please state if this information has not been collected. Report sex- and gender-based analyses where performed, justify reasons for lack of sex- and gender-based analysis.*
Population characteristics	*Describe the covariate-relevant population characteristics of the human research participants (e.g. age, genotypic information, past and current diagnosis and treatment categories). If you filled out the behavioural & social sciences study design questions and have nothing to add here, write "See above."*
Recruitment	*Describe how participants were recruited. Outline any potential self-selection bias or other biases that may be present and how these are likely to impact results.*
Ethics oversight	*Identify the organization(s) that approved the study protocol.*

Note that full information on the approval of the study protocol must also be provided in the manuscript.

Field-specific reporting

Please select the one below that is the best fit for your research. If you are not sure, read the appropriate sections before making your selection.

☒ Life sciences ☐ Behavioural & social sciences ☐ Ecological, evolutionary & environmental sciences

For a reference copy of the document with all sections, see nature.com/documents/nr-reporting-summary-flat.pdf

Life sciences study design

All studies must disclose on these points even when the disclosure is negative.

Sample size	Animal numbers were determined empirically based upon the number of animals typically used for similar experiments in the published literature.<br>iDISCO experiments: 3 males, for verification of staining specificity<br>Barrier experiments: 8 WT males, 7 M5-DTA males (EvansBlue), 3 males (Vimentin staining)<br>Ca2+ imaging: for denatonium the N=68 cell bodies and N=56 processes were pooled from brain slices of 6 different mice, and N=48 cells bodies from 3 different mice for stevioside. Cellular response in Trpm5 knock out experiments were collected from 62 cell bodies for wild type and 43 for the knock-out (3 mice each condition). Representative Ca2+ imaging heatmaps include pooled cells from brain slices of 1-3 mice for each substance. Qualitative images demonstrating Ca2+ responses in brain slices are presented from single coronal sections.<br>RNA-seq experiments: 4 libraries were used for the normal feeding condition and 4 libraries were used for the fasting condition; 3 libraries were used for activation condition and 3 libraries were used for control condition.<br>Stevioside ICV injection and c-Fos staining: 5 male M5-GFP mice were used for saline injection, 9 male M5-GFP mice were used for stevioside injection.<br>Leptin ICV injection and Stat5 staining: 5 male WT mice and M5-GFP mice were used for saline injection, 9 male WT-DTA mice and M5-GFP mice were used for leptin injection. 6 male M5-DTA mice were used for leptin injection.<br>iDTR-mediated ablation test: 7 male mice were used for control injection, 8 male mice were used for iDTR ablation.<br>Metabolic phenotype: 11 WT-iDTR type and 10 M5-iDTR male mice were used.<br>M5-GFP tanycyte counting: 4 M5-GFP male mice were used.<br>M5-DREADD tanycyte activation: 10 male mice were used.<br>Luminex experiment: 8 male mice were used.<br>Glucose tolerance test for virus injected mice: 8 wild type and 5 WT-Dreadd male mice were used.

Data exclusions	no data exclusions
Replication	Reproducibility was verified with three or more (n>=3) replication experiments and all attempts at replication were successful.
Randomization	Mice of the same strain were chosen and allocated randomly. For metabolic and iDISCO experiments male mice were used. For Ca2+ imaging both male and female mice were used.
Blinding	The investigators were blinded to group allocation during data collection. Investigators were aware of the Genotype for allocation in the groups.

Reporting for specific materials, systems and methods

We require information from authors about some types of materials, experimental systems and methods used in many studies. Here, indicate whether each material, system or method listed is relevant to your study. If you are not sure if a list item applies to your research, read the appropriate section before selecting a response.

Materials & experimental systems

n/a	Involved in the study
☐	☒ Antibodies
☐	☒ Eukaryotic cell lines
☒	☐ Palaeontology and archaeology
☐	☒ Animals and other organisms
☒	☐ Clinical data
☒	☐ Dual use research of concern

Methods

n/a	Involved in the study
☒	☐ ChIP-seq
☐	☒ Flow cytometry
☒	☐ MRI-based neuroimaging

Antibodies

Antibodies used

rabbit anti-GFP (1:10000, Invitrogen #A-6455)
goat anti-CD31 (1:5000, R&D Systems, #AF3628)
chicken anti-vimentin (1:2500 (iDISCO), 1:500 (Immunohistochemestry), GeneTex, GTX30668)
chicken anti-mouse PV1 (1:5000, custom-prepared)
rabbit anti-c-Fos (1:500, Cell Signaling Technology, #2250)
chicken anti-GFP (1:1000, ThermoFisher, #A10262)
rabbit anti-pSTAT5 (1:600, Cell Signaling Technology, #9359)
chicken anti-HA tag (1:1000, ThermoFisher, #PA5-33243, AB_2550658)
goat anti-Human HB-EGF (1:1000 R&D System, #AF-259-NA)
rabbit anti DsRed (1:1000, TaKaRa, #632496)

donkey anti-chicken 488 (1:500, Jackson ImmunoResearch, #703-545-155)
goat anti-rabbit Cy3 (1:500, Jackson ImmunoResearch, #711-165-152)
donkey anti-rabbit Cy5 (1:500, Jackson ImmunoResearch, #711-175-152)
donkey anti-goat Cy2 (1:500, Jackson ImmunoResearch, #705-225-147)
donkey anti-goat Cy3 (1:500, Jackson ImmunoResearch, #705-165-147)
donkey anti-chicken 488 (1:500, Jackson ImmunoResearch, #703-545-155)
donkey anti-chicken Cy3 (1:500, Jackson ImmunoResearch, #703-165-155)
goat anti-rabbit cy3 (1:500, Invitrogen, #A10520)
goat anti-chicken Alexa 488 (1:500, Invitrogen, #A11039)

Validation

rabbit anti-GFP (1:10000, Invitrogen #A-6455):
Manufacturer statement: The GFP was isolated directly from the jellyfish Aequorea victoria as immunogen. This Antibody was verified by Relative expression to ensure that the antibody binds to the antigen stated. Species Reactivity: Tag. Antibody detects GFP in Western Blot (WB), immunohistochemistry, immunocytochemistry, flow cytometry, ELISA and immunoprecipitation.

goat anti-CD31 (1:5000, R&D Systems, #AF3628):
Manufacturer statement: Detects mouse CD31/PECAM-1 in direct ELISAs and Western blots. In direct ELISAs and Western blots, approximately 10% cross-reactivity with recombinant human CD31 and recombinant porcine CD31 is observed. Detects mouse CD31 and rat CD31 in flow cytometry

chicken anti-vimentin (1:2500 (iDISCO), 1:1000 (Immunohistochemestry), GeneTex, GTX30668)
Manufacturer statement: Recombinant human Vimentin purified from E. coli. [Swiss-Prot# P08670], tested in WesternBlot.

chicken anti-mouse PV1: directed and affinity-purified against the C-t sequence (CKK)-LPVVNPAAQPSG, custom-prepared by Caslo, Kongens Lyngby, Denmark; labeling identical to that given by the rat monoclonal clone MECA-32 in Ciofi et.al (Ciofi P. The arcuate nucleus as a circumventricular organ in the mouse. Neurosci Lett 487, 187-190 (2011)).

rabbit anti-c-Fos (1:500, Cell Signaling Technology, #2250)
Manufacturer statment: This antibody detects endogenous levels of total c-Fos protein. The antibody does not cross-react with other Fos proteins, including FosB, FRA1 and FRA2. Tested in immunofluorescence. Species Reactivity: Human, Mouse, Rat.

chicken anti-GFP (1:1000, ThermoFisher, #A10262)
Manufacturer statment: This Antibody was verified by Relative expression to ensure that the antibody binds to the antigen stated. Tested in immunofluorescent analysis. Species Reactivity: Tag

rabbit anti-pSTAT5 (1:600, Cell Signaling Technology, #9359)
Manufacturer statment: Phospho-Stat5 (Tyr694) (C11C5) Rabbit mAb detects endogenous levels of Stat5a only when phosphorylated at Tyr694 and Stat5b when phosphorylated at Tyr699. Tested in immunohistochemical analysis. Species Reactivity: Human, Mouse.

chicken anti-HA tag (1:1000, ThermoFisher, #PA5-33243, AB_2550658)
Manufacturer statment: Chicken anti influenza heamagglutinin antibody recognizes influenza hemagglutinin, a glycosylated antireceptor which binds to cell surface glycoproteins prior to penetration of the cell membrane and uncoating of the virus. Synthetic peptide YPYDVPDYA from influenza hemagglutinin (HA) was used as immunogen. Species Reactivity: Tag. Antibody detects HA tag in Western Blot, Immunohistochemistry (Frozen)(IHC(F)) and Immunoprecipitation.

goat anti-Human HB-EGF (1:1000 R&D System, #AF-259-NA)
Manufacturer statment: Detects human HB-EGF in ELISAs and Western blots. In direct ELISAs, less than 1% cross reactivity with recombinant mouse HB-EGF is observed. In sandwich immunoassays, less than 0.1% cross-reactivity with recombinant human (rh) Amphiregulin, rhBetacellulin, rhEpiregulin, and recombinant mouse Epigen is observed.

rabbit anti DsRed (1:1000, TaKaRa, #632496)
Manufacturer statment: The Living Colors DsRedPolyclonal Antibody has been raised against DsRed-Express, a variant ofDiscosoma sp.redfluorescent protein. This antibody recognizes DsRed-Express, DsRed-Express2, mCherry, DsRed2, E2-Crimson,tdTomato, mStrawberry, and mBanana, and both N-and C-terminal fusion proteins containing these fluorescent proteins in mammalian cell lysates. Used for immunoprecipitation and immunolabeling applications in numerous studies.

Eukaryotic cell lines

Policy information about cell lines and Sex and Gender in Research

Cell line source(s)	HEK 293T cells were obtained from Sigma.
Authentication	Cell lines were not authenticated.
Mycoplasma contamination	Cell lines were tested for mycoplasma contamination and they were negative.
Commonly misidentified lines (See ICLAC register)	No cell line used in the paper is listed in ICLAC database.

Animals and other research organisms

Policy information about studies involving animals; ARRIVE guidelines recommended for reporting animal research, and Sex and Gender in Research

Laboratory animals	Mus musculus, mixed background<br>iDISCO experiments: M5-GFP mice, males, 10-14 weeks of age<br>EvansBlue: WT-DTA and M5-DTA mice, 15 males, 10-14 weeks of age<br>Vimentin immunohistochemistry: M5-GFP mice, 3 males, 10-14 weeks of age<br>Ca2+ imaging: M5-GCaMP mice, males and females 10-24 weeks of age<br>RNA-seq experiments: M5-GFP and M5-DREADD-GFP mice, 28 12 -16 week-old male mice were used.<br>stevioside ICV injection and c-Fos staining: M5-GFP mice, male 12-16 weeks of age<br>leptin ICV injection and Stat5 staining: WT, WT-DTA, M5-GFP and M5-DTA mice, male 12-16 weeks of age<br>Metabolic study and iDTR ablation: M5-GFP-iDTR, M5-iDTR and WT-iDTR mice 5 weeks of age; phenotyping: M5-iDTR and WT-iDTR mice 7-48 weeks of age<br>Tanycyte counting: M5-GFP mice, male 12-16 weeks of age<br>TRPM5-DREADD tanycyte activation for glucose tolerance test: WT-GFP/DREADD, WT and WT-DREADD mice, 12-16 weeks of age<br>Luminex experiment: M5-DREADD, WT-DREADD mice, 12-16 weeks of age
Wild animals	no wild animals.
Reporting on sex	Findings not apply to only one sex. Sex was not considered in study design.
Field-collected samples	no field-collected samples
Ethics oversight	Welfare committee of Saarland University (permission for perfusion and organ collection AZ: GB3-2.4.7.1-Boe)<br>Welfare committee of Saarland University and Regierung Oberbayern (22/2018)<br>French Ministry of National Education, Higher Education and Research (APAFIS#2617-2015110517317420 v5)

Note that full information on the approval of the study protocol must also be provided in the manuscript.

Flow Cytometry

Plots

Confirm that:

☒ The axis labels state the marker and fluorochrome used (e.g. CD4-FITC).

☒ The axis scales are clearly visible. Include numbers along axes only for bottom left plot of group (a 'group' is an analysis of identical markers).

☒ All plots are contour plots with outliers or pseudocolor plots.

☒ A numerical value for number of cells or percentage (with statistics) is provided.

Methodology

Sample preparation	Adult male TRPM5-IC/eR26-τGFP and TRPM5-IC/eR26-τGFP/DREADD mice were i.c.v. injected with AAV2/1+2-CAGS-mCherry virus three weeks before cell sorting. The hypothalamus from mice, which either underwent 14 hours of fasting or normal diet feeding, or injected with CNO into 3rd ventricle were dissected and pooled from 2 mice. After dissection, the hypothalamus were cut into small pieces and placed in a solution of papain and deoxyribonuclease in Earle's balanced salt solution (Papain dissociation system; Worthington) and incubated for 30 minutes with gentle agitation at 37°C. Afterward, the tissue was dissociated by repeated pipetting with a fire-polished glass pipette. The dissociated cells were then transferred to a 15-mL Falcon tube and centrifuged at 1000 rpm for 5 minutes. The supernatant was then discarded and the cell pellet resuspended in 1 mL of FACS buffer (0.1% BSA, 1% EDTA and 50ng/ml Hoechst 33258 dye in PBS). The dissociated cells were then sorted using FACS (Sony SH800).
Instrument	Sony SH800
Software	SH800S software version 2.1.5
Cell population abundance	Approximately 1000 sorted M5 tanycytes for two hypothalamus were obtained
Gating strategy	FSC and SSC were used firstly to determine the cells and tissue debris, then the area versus the height of FSC was used for doublet discrimination. In the end, cells were sorted by fluorescence (endogenously expressed GFP and mCherry from virus) with excitation at 488 nm and 561 nm, and emission was detected in FL2 (525/50 nm) and FL3 (600/60 nm).

☒ Tick this box to confirm that a figure exemplifying the gating strategy is provided in the Supplementary Information.
